# Plastid phylogenomics sheds light on divergence time and ecological adaptations of the tribe Persicarieae (Polygonaceae)

**DOI:** 10.3389/fpls.2022.1046253

**Published:** 2022-12-08

**Authors:** Dong-Ling Cao, Xue-Jie Zhang, Xiao-Jian Qu, Shou-Jin Fan

**Affiliations:** Shandong Provincial Key Laboratory of Plant Stress Research, College of Life Sciences, Shandong Normal University, Ji’nan, China

**Keywords:** Persicarieae, plastome, ecological adaptations, divergence time, phylogeny, morphological characters

## Abstract

Southwestern China, adjacent to the Qinghai-Tibetan Plateau (QTP), is known as a hotspot for plant diversity and endemism, and it is the origin and diversification center of Persicarieae. As one of the major lineages in Polygonaceae, Persicarieae represents a diverse adaptation to various habitats. As a result of morphological plasticity and poorly resolving molecular markers, phylogenetic relationships and infrageneric classification within Persicarieae have long been controversial. In addition, neither plastome phylogenomic studies nor divergence time estimates on a larger sample of Persicarieae species have been made thus far. We sequenced and assembled 74 complete plastomes, including all of the recognized genera within Persicarieae and their relatives. We conducted a comprehensive phylogenetic study of the major clades within Persicarieae and, based on the thus obtained robust phylogeny, also estimated divergence time and the evolution of diagnostic morphological traits. Major relationships found in previous phylogenetic studies were confirmed, including those of the backbone of the tree, which had been a major problem in previous phylogenies of the tribe. Phylogenetic analysis revealed strong support for *Koenigia* as sister to *Bistorta*, and together they were sister to the robustly supported *Persicaria*. Based on the phylogenetic and morphological evidence, we recognize five sections in *Persicaria*: *Persicaria*, *Amphibia*, *Tovara*, *Echinocaulon*, and *Cephalophilon*. It is estimated that the divergence of the Persicarieae began around the late Paleocene, with diversification concentrated in the Eocene and Miocene. In addition, it is suggested that the increasing westerly and monsoon winds in conjunction with the uplift of the QTP may be the driving force for origin and diversification of Persicarieae species. These results provide a valuable evolutionary framework for the study of adaptation in Polygonaceae and insights into plant diversification on the QTP and adjacent areas.

## Introduction

Polygonaceae is composed of annual or perennial herbs or vines, mainly distributed in the northern temperate and tropical regions (Sanchez et al., 2009; Schuster et al., 2011a; Schuster et al., 2011b). Many Polygonaceae species are economically important, such as those used in traditional Chinese medicine (e.g., *Fallopia*), as horticultural ornamentals (e.g., *Reynoutria*), and in the development of the ecological environment (e.g., *Calligonum* and *Persicaria*) (Sun et al., 2014a; Olaru et al., 2015; Choi et al., 2019; Fan et al., 2021; Zhang et al., 2021). Due to the high diversity of Polygonaceae, large‐scale phylogenies have been used to address questions on taxonomy and ancestral states (Frye and Kron, 2003; Sanchez et al., 2009; Sanchez et al., 2011; Schuster et al., 2015). However, the inadequate level of informative sites in the molecular data have often led to poor phylogenetic resolution and inconsistent species delineation.

As one of the major groups within Polygonaceae, the diversity of Persicarieae species is a classic example of ecological adaptations to various habitats (Li et al., 2003; Fan et al., 2013; Sun et al., 2014a). Persicarieae are a group of predominately herbaceous plants distributed worldwide, showing different species densities between 0 and 5000 m in altitude (Sanchez et al., 2009; Sanchez et al., 2011). For example, members of *Koenigia* (~20 species) have been recorded in the Himalayas and neighboring alpine regions of the Hengduan Mountains (HDM) in southwestern China (Zhou et al., 2004; Schuster et al., 2015; Sun et al., 2017) and have the potential to provide insight into the biogeographic diversity of eastern Himalayan plants (Zhou et al., 1999a; Zhou et al., 2004; Xu et al., 2009). *Koenigia islandica*, for instance, is a keystone species in alpine ecosystems, with a wide distribution in the Arctic and alpine regions of the Northern Hemisphere and even in southern South America, demonstrating a bipolar division. *Bistorta*, which includes many species in the alpine regions, is an appropriate model for examining ecological adaptations in alpine-cold habitats. For example, in *B. vivipara*, the sexual reproductive (flower) allocation increases as elevation increases, which is an adaptive response that improves sexual reproductive success in harsh alpine environments (Qi et al., 1998; Fan and Yang, 2009; Sun et al., 2014a). Members of the genus *Persicaria* have a high degree of adaptability and plasticity to the aquatic environment (Li et al., 2003; Qu and Xu, 2015; Wei, 2019). For example, *P. hydropiper* can respond to flood stress by growing bigger and by thickening its leaves, thus reducing input to reproductive growth (Wei, 2019).

An excellent region to study such questions is southwestern China, adjacent to the Qinghai-Tibetan Plateau (QTP), known as a hotspot for plant diversity and endemism. It is the origin and diversification center for Persicarieae (Zhou et al., 1999a; Zhou and Li, 2003). The periglacial region of the HDM in southwest China has the richest flora of any habitat of its kind in the world (Hu et al., 2021; Xia et al., 2022). The study of trait evolution and ecological adaptations within the framework of time divergence has been a topic of research interest (Mao et al., 2021; Xu and Chen, 2021). Numerous hypotheses, theories, and conclusions have been proposed (Frye and Kron, 2003; Schuster et al., 2013). In the harsh natural environment, studying the origin and evolution of biodiversity and its response to environmental changes can not only elucidate the process of forming plant diversity but also predict the impact of future climate change on alpine plant diversity.

Molecular data revealed a significant advance in our understanding of evolutionary relationships of major groups within Persicarieae (Kim and Donoghue, 2008a; Schuster et al., 2011b; Schuster et al., 2015); however, the results are inconsistent with previously recognized delimitation based on morphological evidence, particularly for clades with complex evolutionary histories. Whereas there is strong evidence for the monophyly of Persicarieae (Schuster et al., 2013; Schuster et al., 2015), the delimitation of several contentious subclades remains particularly problematic. Although members of the genus *Persicaria* are usually easily recognized by the presence of an ocrea, nodes that are swollen in comparison to the axis, and quincuncial aestivation, some subgroups are difficult to identify because of overlapping character states and morphological plasticity (Decraene and Smets, 2000; Choudhary et al., 2012; Fan et al., 2013; Paul and Chowdhury, 2021). Earlier studies considered *Aconogonon*, *Bistorta*, *Koenigia*, and *Persicaria* to be closely related genera, with roughly 200 known species worldwide (Sanchez et al., 2009; Sanchez et al., 2011). Traditional systematic taxonomic studies have classified species within *Persicaria* in a narrow sense into different sections based on morphology (Li et al., 2003; Hou, 2006). However, some molecular studies suggest that these sections should be treated as genera and the relationships between these new genera should be re-established (Li et al., 2004; Zhao et al., 2012). A series of molecular and morphological studies support a close phylogenetic relationship between *Koenigia* and *Aconogonon*, but there is no consensus on the delineation and classification of this clade (Fan et al., 2013; Schuster et al., 2015). Most studies usually focus only on one or two representatives per clade, and phylogenetic relationships within the clades are correspondingly poorly known (Li et al., 2003). Moreover, the phylogenetic position of some controversial species (i.e., *K. cyanandra*, *P. longiseta*, *P. lapathifolia* var. *salicifolia*, and *P. bungeana*) is in dispute (Liu et al., 2007; Min et al., 2013).

The complete plastome contains enough sequence variation to allow phylogenetic relationships between closely related species to be resolved, including species that have experienced complex evolutionary histories (Xie et al., 2019; Zhou et al., 2020). Indeed, complete plastomes have significantly advanced our understanding of the evolutionary relationships of the other major clades of Polygonaceae (Sun et al., 2012; Zhang et al., 2014b; Fan et al., 2021). The use of the complete chloroplast sequences to reconstruct phylogenetic relationships has made it possible to identify evolutionary histories, including character state evolution, while also revealing previously unrecognized levels of species diversity (e.g., *K. delicatula*, *P. bungeana*, and *B. emodi*). Thus far, the plastomes of only a few *Bistorta* and *Koenigia* species have been sequenced. Several researchers have focused on the diversification of taxa in different distribution areas and have put forward hypotheses of character evolution (Decraene and Akeroyd, 1998; Zhou et al., 1999b; Frye and Kron, 2003; Zhou et al., 2004), but only few studies emphasize the response of adaptive characters to climate change. Besides the controversy concerning our understanding of phylogenetic relationships, a dated phylogeny allowing the radiation history of Persicarieae to be elucidated is still lacking.

Here, we reconstruct the phylogenetic relationships of Persicarieae, including all currently recognized genera and contentious closely related taxa. The objectives were (1) to clarify the phylogenetic relationship of some controversial clades within Persicarieae; (2) to investigate the evolution of key morphological characters; and (3) to estimate divergence times of major Persicarieae clades allowing evolutionary events to be put in a temporal context.

## Materials and methods

### Taxon sampling

To reconstruct a robust phylogeny of Persicarieae, we sampled all genera recognized to date in Persicarieae, as well as controversial closely related taxa (Schuster et al., 2015). A total of 78 plastome sequences, representing 59 individuals of Persicarieae, seven individuals of Fagopyreae, and 12 individuals of Polygoneae, were included, 72 of those newly generated in this study. Voucher information for each sample is provided in **Table S1**. In addition, to confirm species identity and evaluate whether there are obvious differences in plastomes from different locations, two individuals of each species considered taxonomically controversial were sampled from different locations (such as *P. amphibia* 1 and *P. amphibia* 2). Individuals of the representative species were collected in the field or were taken from herbarium vouchers already present in Shandong Normal University.

### DNA sequencing, assembly, annotation, and comparative analysis

Our samples were obtained from silica dried tissues or herbarium material. Voucher specimens were deposited in the herbarium of Shandong Normal University. Source details of all 72 individuals are provided in **Table S1**. Total genomic DNA was extracted from silica‐dried leaves and herbarium specimens using a modified CTAB method (Allen et al., 2006). About 1.5 µg of high‐quality DNA from each of the samples was used to build shotgun libraries with 350 bp insert sizes. Genomic DNA was sequenced using an Illumina MiSeq2000 sequencer following the manufacturer’s protocol (Illumina, CA, USA). Sequencing in 150-bp paired-end mode was conducted on the Illumina Novaseq platform at Novogene (Tianjin, China). Complete plastomes were assembled using both GetOrganelle 1.7.0 (Jin et al., 2020) and SPAdes 3.14.1 (Bankevich et al., 2012). Plastomes were annotated with Plastid Genome Annotator (Qu et al., 2019) and then manually inspected to correct unclear sections with Geneious 8.0.2 (https://www.geneious.com). Organellar Genome DRAW tool 1.3.1 was used to draw the circular chloroplast genome maps (Greiner et al., 2019). All newly obtained plastomes were submitted to GenBank. To compare the plastomes full alignments with annotations were visualized using Shuffle‐LAGAN mode in mVISTA software (Frazer et al., 2004) with the default settings and *P. amphibia* as a reference sequence. Simple sequence repeats (SSRs) were predicted by the microsatellites identification tool MISA (http://pgrc.ipk‐gatersleben.de/misa/) with the parameters set to at least 10 repeat units for mononucleotide SSRs, at least six repeat units for dinucleotide, at least five repeat units for trinucleotide, at least four repeat units for tetranucleotide, and at least three repeat units for pentanucleotide and hexanucleotide motifs. Repetitive sequences were identified using the REPuter program (Kurtz et al., 2001; http://genome.lbl.gov/vista/index.shtml) where Forward (F), Reverse (R), Palindromic (P), and Complementary (C) repeats were identified following the procedure of Ni et al. (2016). The minimum repeat size was set to eight, and duplicated sequences in the IR region were excluded. To detect the expansion or contraction of the inverted repeat (IR) region among Persicarieae, we used IRscope (Amiryousefi et al., 2018) to visualize the borders of the LSC, SSC, and IR.

### Sequence alignment and phylogenetic analyses

To determine sequence divergence among Persicarieae plastomes, we undertook a sliding window analysis to calculate nucleotide diversity (π) using DnaSP 6.12.03 (Liu et al., 2019). The screening conditions were as follows: (1) sequence length > 200 bp; (2) variable sites and parsimony information sites > 0. Phylogenetic analyses were undertaken on the 80 complete plastome sequences using maximum likelihood (ML) and Bayesian inference (BI), with *Rumex maritimus* and *R. nepalensis* as outgroups. Custom Perl scripts were used to extract all coding and noncoding sequences from each plastome (https://www.github.com/quxiaojian/BioinformaticScripts/extract sequence from gb file). In total, 79 protein-coding genes (PCGs) and 130 noncoding sequences were individually aligned using MAFFT 7 (Katoh and Standley, 2013) and corrected manually using Geneious 8.0.2. Sequences were aligned visually. As there were no bad sequences, we did not perform further filtration. We combined the two datasets, sequences and coded indels, using the “concatenation” option in Geneious 8.0.2. The phylogeny was reconstructed using ML and BI, as these are less sensitive to long-branch attraction than parsimony methods (Bergsten, 2005). Each gene partition was tested for the best‐fit substitution model using jModelTest 0.1.1 (Posada, 2008). Considering that different plastome regions have distinct molecular evolutionary rates (Zhu et al., 2016), both ML and BI were constructed for the following data sets: (1) 79 PCGs; (2) 130 noncoding sequences; (3) complete plastomes. The ML analyses were implemented in RAxML 8.2.8 (Stamatakis, 2014) with 1000 bootstrap replicates using the GTR + G model. The same model was used in a BI analysis with MrBayes 3.2.6 (Ronquist and Huelsenbeck, 2003). Two Markov Chain Monte Carlo (MCMC) searches were run with four chains each for 10 million generations each. The sampling frequency was every 1000 generations, and the first 25% were discarded as burn-in. Tree congruence was assessed by visually comparing topologies and support values, and no mutually well-supported conflicts were observed. Text and graphics provide clade support percentages using percent BS (bootstrap support) and percent PP (Bayesian posterior probability), such as 98/1 (BS/PP). For the coalescent method, individual gene trees for each of 79 PCGs were inferred by RAxML and 1000 rapid bootstrap replicates, and the species tree was reconstructed by ASTRAL-III (Zhang et al., 2018). The final phylogenetic topologies were visualized using Figtree 1.3.1.

### Principal component analysis and ancestral state reconstruction

State data for the morphological characteristics were collected from herbarium specimens, experimental observation data, our field observations, and from previous publications (Decraene and Smets, 2000; Li et al., 2003; Kong and Hong, 2019; Kong et al., 2021; Paul and Chowdhury, 2021). Immersion in warm water was used to rehydrate the dry sample, which was then inspected under the dissecting microscope, the light microscope (LM), and the scanning electron microscope (SEM). Detailed descriptions and evaluations of the various traits are presented in **Table S2**. Principal component analysis (PCA) was performed in Origin (2021) software (Origin Lab Corporation, Northampton, MA, USA) using seven characteristics (leaf venation, stomatal type, epidermal cell shape, inflorescence type, tepal venation, structure of exocarp slices, and fruit surface ornamentation) of 78 species. In Persicarieae, there are two types of leaf venation: palmate venation and pinnate venation. The stomata are anisocytic, anomocytic, paracytic, or a mixture of both. Generally, the type of tepal venation is 3-basinerved and pinnate. We describe the shape of the abaxial epidermal cells of the leaves, as either polygonal or irregular. According to the shape of the cavity in the exocarp, it can be divided into dichotomous, dichotomous to trichotomous, absent, or a mixture of these types of branching. The morphological characters used for the analysis are detailed in **Table S2**.

For ancestral character state reconstruction, we focused on characters considered to be diagnostic in previous taxonomic treatments (i.e., pollen type, tepal number, inflorescence type, style number, and stamen number). Specifically, there are five types of pollen apertures (3-colpate, polyplicate, pantocolpate, pantoporate, and 3-colporate), three types of perianth merosity (three-, four-, and five-merous), four inflorescences types (raceme, panicle, capitulum, and cyme), two classes of style numbers (three in most species, with only a few having two), and three classes of stamen numbers (the majority has eight, with only a few species have five or six). The ML function in Mesquite 2.74 (Maddison and Maddison, 2007) was used to trace character state evolution on the best tree obtained from ML analysis of the complete plastome dataset.

### Estimates of divergence times

The penalized likelihood, a relaxed clock method, was used to conduct a dating analysis by using treePL (Smith and O’Meara, 2012). A cross-validation method was applied to acquire the optimal value for λ by testing 13 smoothing values in multiples of 10 from 1×10^-6^ to 1×10^6^ (Sanderson, 2002). A total of 1000 ML bootstrap trees with branch lengths were generated by RAxML and used to estimate the minimum and maximum ages of the internal nodes. According to previous studies (Manchester and O’Leary, 2010; Sun et al., 2012; Schuster et al., 2013; Zhang et al., 2014b; Yao et al., 2019; Zhang et al., 2021), two fossil calibrations and three secondary calibrations were used. The crown age of Polygonaceae was set to an age range of 72.1-66 Ma implemented *via* a lognormal prior distribution. The minimum age of the crown node of *Persicaria*, *Bistorta*, and *Koenigia* was constrained to 55.8 Ma (Nowidke and Skvrla, 1977; Muller, 1981), and the maximum age was set to 77.5 Ma (Schuster et al., 2013). The ML phylogram from the concatenated 79-PCG alignment was used as input. TreeAnnotator 2.6.3 (Drummond et al., 2012) was used to map ages and their confidence intervals obtained from the dated bootstrap trees to the dated ML tree.

## Result

### Genome features

The GC content of all the plastomes was relatively conserved at 37.2-38.2% and genome size ranged from 138,394 bp (*K. delicatula*) to 161,117 bp (*B. emodi*). The length varied from 4,154 to 13,453 bp in the SSC region, 77,434 to 85,853 bp in the LSC region, and 28,403 to 31,216 bp in the IR region (**Table S3**). The majority of plastomes of Polygonoideae contained 79 protein-coding genes, 4 rRNA genes, and 30 tRNA genes arranged in the same gene order except in *K. delicatula* (loss or pseudogenization of all 11 *ndh* genes). The IR/SC boundaries were conserved among most of the examined species, with a few expansions and contractions detected (**Figure S1**). For example, some genes including *rps3*, *rpl22*, *rps19*, *rpl2*, *ndhF*, *rps15*, *ycf1*, *trnH*, and *psbA* were found at the LSC/IR and SSC/IR boundaries.

### Repetitive sequences and nucleotide diversity

Using the microsatellite identification tool MISA, 2093 SSRs were detected in the Persicarieae species (**Table S4**). The majority of SSRs were located in the LSC. We identified 23 (*P. japonica*) to 50 (*B. emodi*) SSRs per plastome consisting of mono- to tri-nucleotide repeats. Most of these SSRs were mononucleotide A/T motifs, followed by dinucleotide motifs predominantly of AT/TA, and trinucleotide repeats with a predominant motif of ATA/ATT/TAT/TTA. The number of oligonucleotide repeat types varied among the 59 cp genomes, but most repeat sequences existed in the range from 30 to 35 bp (**Table S5**). The abundance of Forward and Palindromic repeats was higher than that of Reverse and Complementary repeats. The lowest number of repeats was found in *K. delicatula* (13), whereas the highest was found in *B. amplexiculis* and *B. emodi* (48). **Table S4** contains all of the pertinent information. Persicarieae plastomes sequence identities were plotted using mVISTA, with *P. amphibia* used as a reference (**Figure S2**). The results revealed high sequence similarity among all sequences, suggesting that Persicarieae plastomes are conserved. Eight intergenic spacers with high nucleotide diversity were detected (>0.13); these were *psaJ-rpl33*, *psbK-psbI*, *ndhE-ndhG*, *trnS-GCU-trnG-UCC*, *trnW-CCA-trnP-UGG*, *trnS-GGA-rps4*, *trnH-GUG-psbA*, and *trnG-GCC-trnfM-CAU*. Additionally, four genes (*ccsA*, *ndhJ*, *matK*, and *rps15*) with nucleotide diversity of more than 0.06 were identified (**Figure S3**).

### Phylogenetic reconstruction of Persicarieae

**Figure 1:**
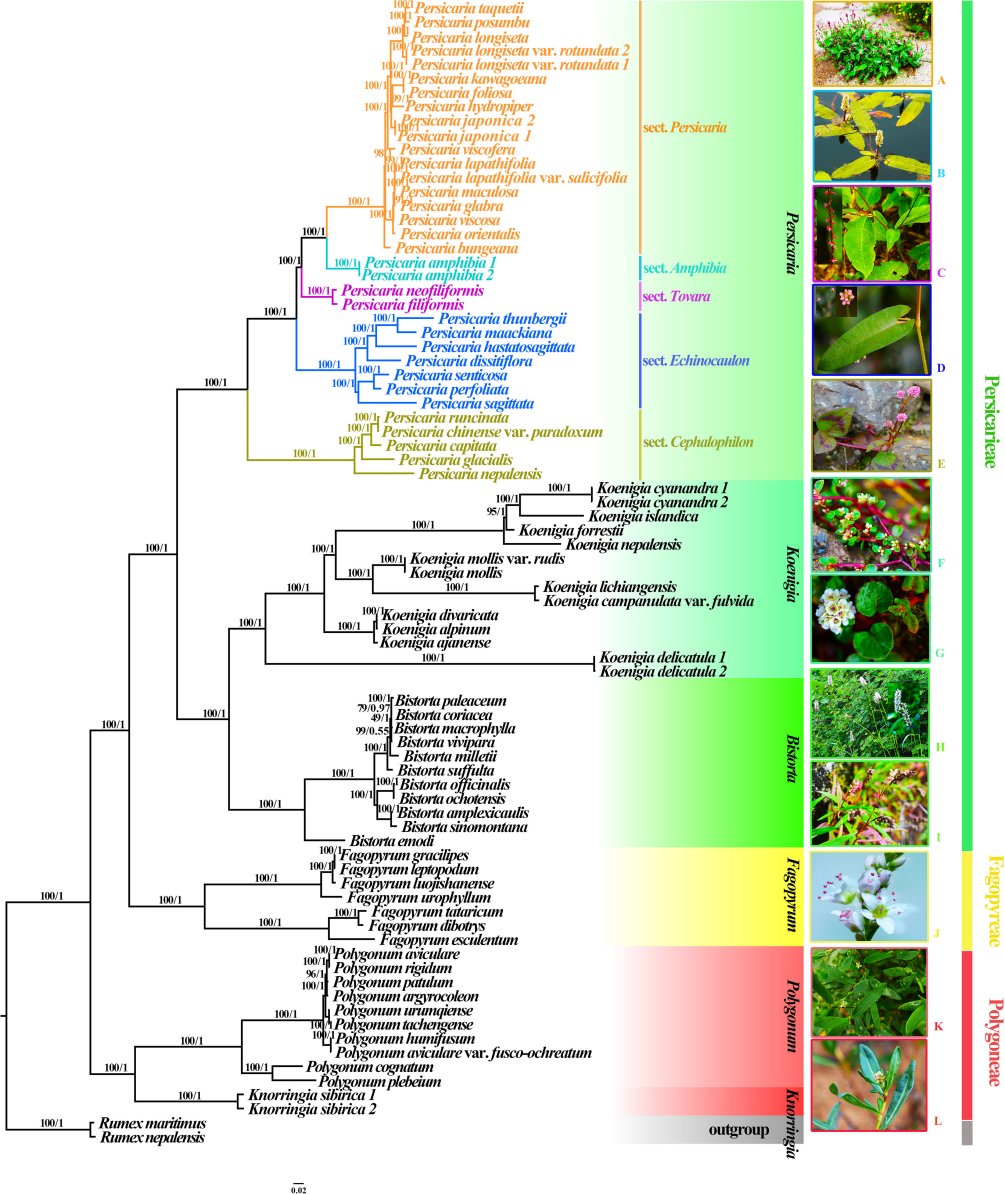
ML and BI phylogenetic trees based on complete plastomes of 80 samples. *Rumex maritimus* and *R. nepalensis* were set as the outgroup, major clades are indicated by different colors. Numbers above each branch indicate ML bootstrap values (BS) and BI posterior probabilities (PP). **(A)**, *Persicaria posumbu* (sect. *Persicaria*); **(B)**, *P. amphibia* (sect. *Amphibia*); **(C)**, *P. filiformis* (sect. *Tovara*); **(D)**, *P. sagittata* (sect. *Echinocaulon*); **(E)**, *P. capitata* (sect. *Cephalophilon*); **(F)**, *Koenigia islandica*; **(G)**, *K. forrestii*; **(H)**, *Bistorta officinalis*; **(I)**, *B. emodi*; **(J)**, *Fagopyrum esculentum*; **(K)**, *Polygonum aviculare*; **(L)**, *Knorringia sibirica*. Photo credits: Dong-Ling Cao and Cai-Cai Zhai.

The alignment of the complete plastome extracted from the 80 accessions was 161,268 bp in length, including variable sites (44,901) and parsimony-informative sites (38,673). All these analyses supported the monophyly of Persicarieae (BS/PP=100/1). The phylogenetic analyses based on complete plastome data (**Figure 1**), CDS (**Figure S4**), and intergenic spacer (**Figure S5**) data resulted in similar topologies. Hence, we used ML trees to illustrate the phylogeny and to show the BS and PP values (**Figure 1**). The ML phylogenetic trees reconstructed from the complete plastomes as well as the ASTRAL coalescent tree (**Figure S6**) yielded highly similar results. The results revealed three strongly supported clades corresponding to Persicarieae, Fagopyreae, and Polygoneae. The backbone of the ML phylogenetic tree reconstructed from the complete plastome analysis of the Persicarieae tree was well resolved. Our study confirmed the well-established relationships of Persicarieae being composed of the three lineages *Persicaria*, *Koenigia*, and *Bistorta* (**Figure 1**). Likewise, there was strong support for a monophyletic *Persicaria* and its sister relationship with a well-supported clade including *Koenigia* and *Bistorta*. The five subclades within *Persicaria* represented the five sections, namely, *Persicaria*, *Tovara*, *Amphibia*, *Echinocaulon*, and *Cephalophilon* each (BS/PP=100/1).

### Morphological trait comparison and ancestral state reconstruction

**Figure 2:**
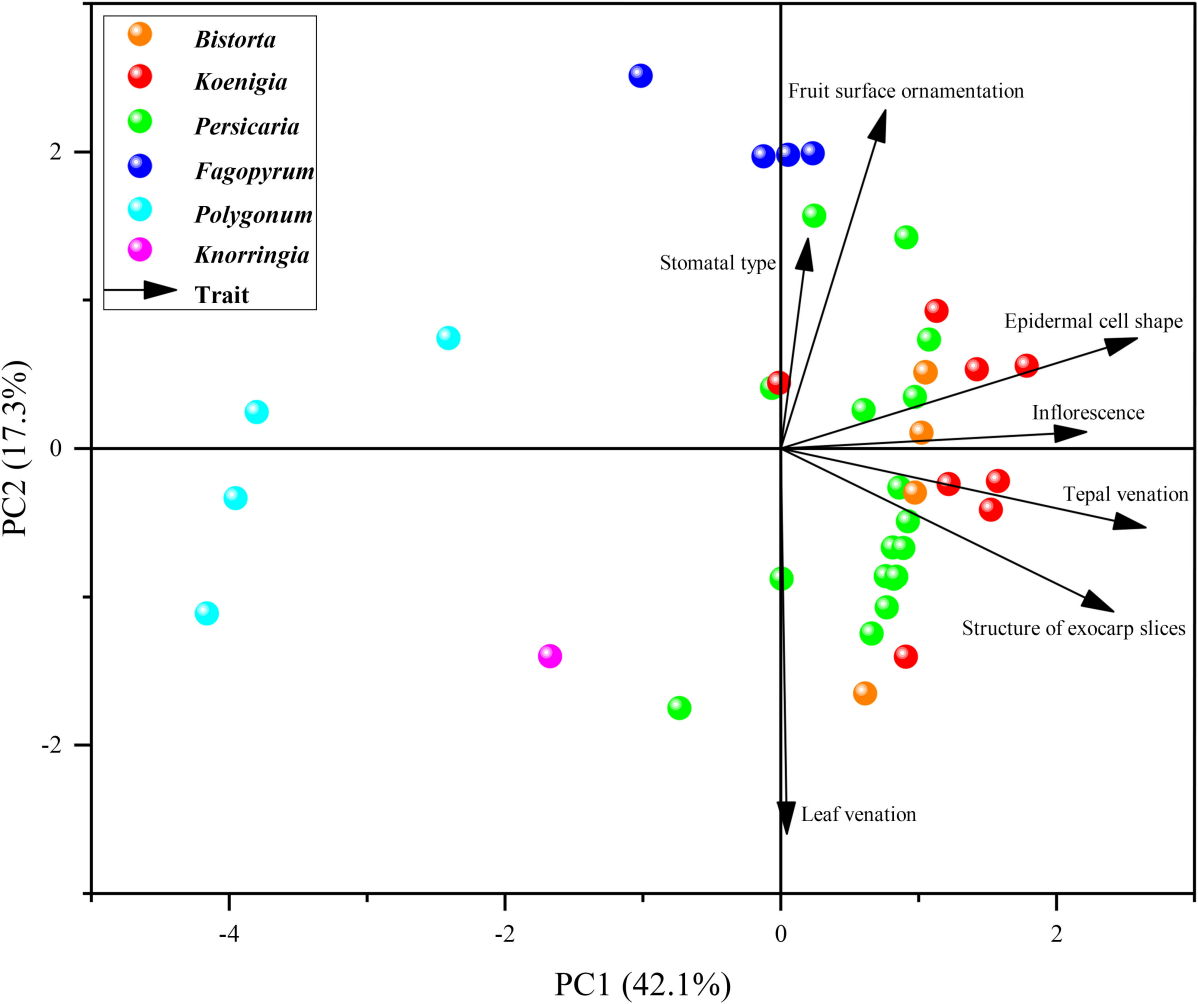
Principal component analysis (PCA) performed with qualitative morphological characters. The solid circles of different colors represent species of different genera. The black arrows represent the seven characters.

PCA divided the characters into three groups, Persicarieae, Fagopyreae, and Polygoneae (**Figure 2**). The distribution of total variation in PC1 and PC2 was 42.1% and 17.3% respectively. The species of *Bistorta*, *Koenigia*, and *Persicaria* clustered together (**Figure 2**). *Bistorta* and *Koenigia* taxa were found nested within *Persicaria*.

**Figure 3:**
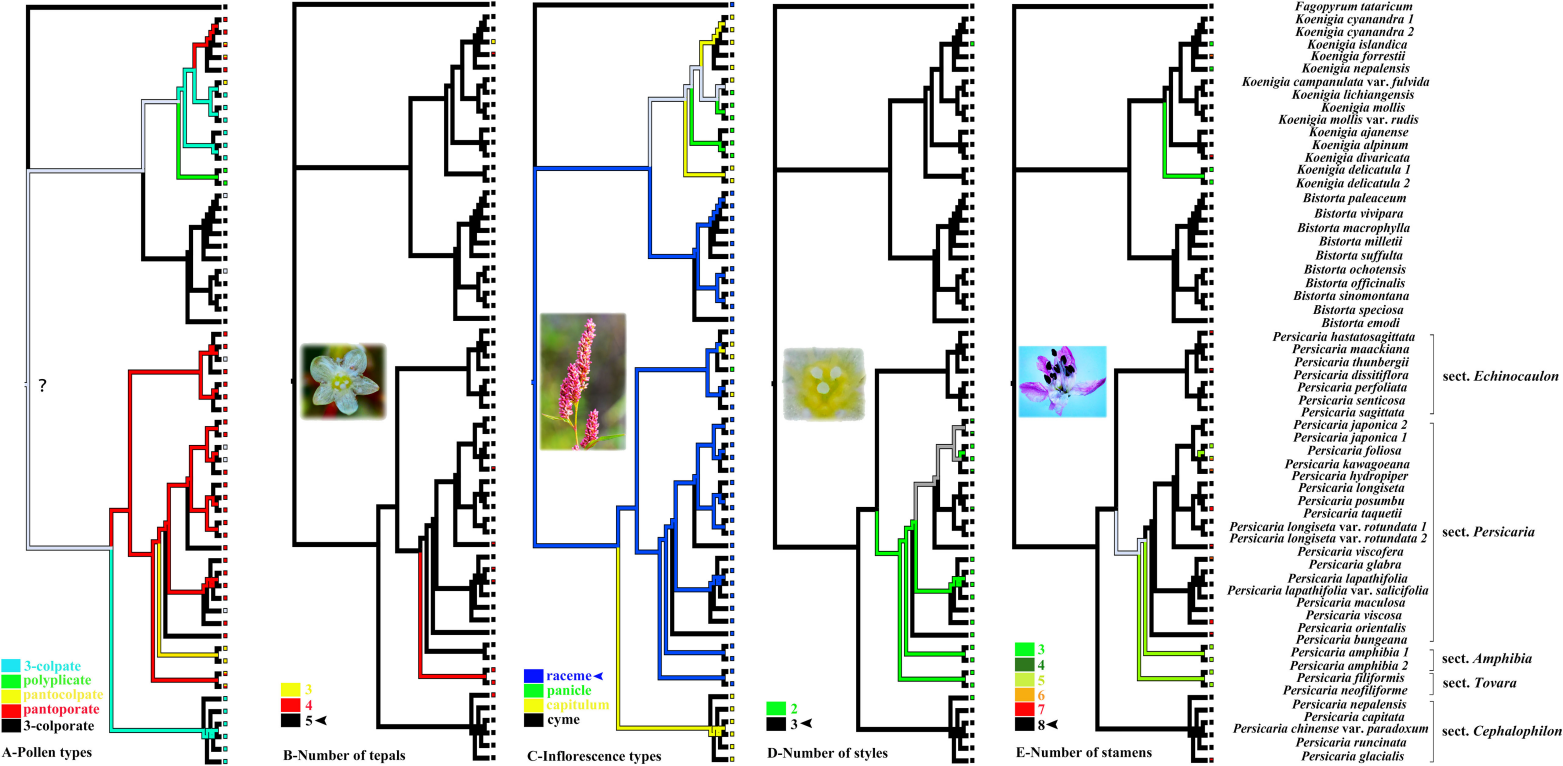
Ancestral state reconstruction of pollen types, the number of tepals, inflorescences types, the number of styles and stamens based on relationships implied by the RAxML tree. The ink line diagram represents the reconstructed ancestral state. The question mark indicates uncertainty. Photo credits: Dong-Ling Cao.

The evolutionary history of pollen is slightly difficult to infer because the ancestral state of the tribe was still unclear (**Figure 3A**). The five-merous perianth was inferred as the ancestral state and thus was pleiomorphic in the tribe, whereas three or four merous perianth were derived (**Figure 3B**). Raceme was reconstructed as the ancestral state in the clade of Persicarieae, but the transition to a capitulum likely occurred early in the evolution of *P.* sect. *Cephalophilon* (**Figure 3C**). In Persicarieae, three styles and eight stamens were inferred as ancestral states, deviating numbers (two styles and five to six stamens) were derived (**Figures 3D, E**).

### Estimation of divergence times

**Figure 4:**
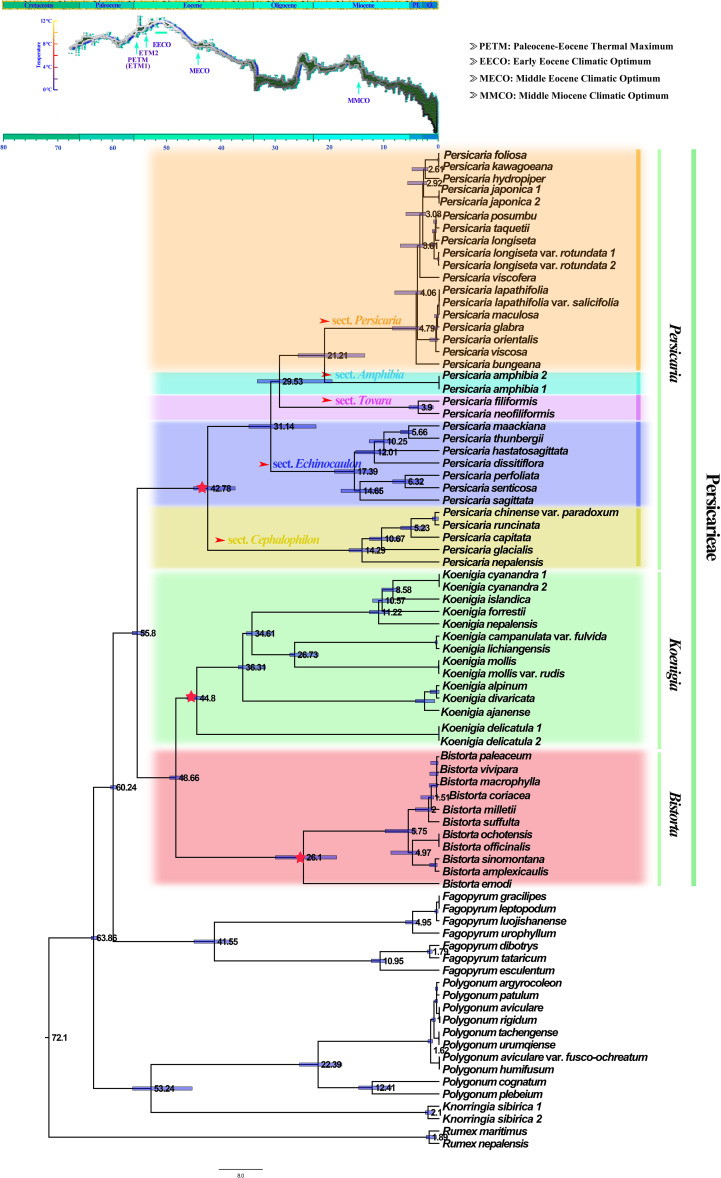
Chronogram of Persicarieae obtained with TreePL. Blue bars span from the minimum to the maximum estimates of the age of each internal node. The number at the node represents the age of the clade. The three pentagrams represent the divergence times of the three genera in Persicarieae. The five arrows point to the divergence times of the five sections within *Persicaria*. The chronogram used a deep-sea benthic foraminiferal oxygen-isotope curve as modified by Zachos et al. (2019), showing the evolution of global climate over the last 70 Ma. The ages of stratigraphic boundaries are from the International Chronostratigraphic Map (Pl., Pliocene; Q, Quaternary).

Persicarieae and other Polygonaceae species diverged approximately 55.8 (95% highest posterior probability density [HPD]: 55.8-57.8 Ma) million years ago (Ma), close to the Paleocene-Eocene Thermal Maximum (ETM1). *Koenigia* and *Bistorta* diverged from other Persicarieae ca. 44.80 Ma (95% [HPD]: 43.27-46.60 Ma) and 26.10 Ma (95% HPD: 18.97-30.29 Ma), respectively. Crown age of *Persicaria* was estimated to be ca. 42.78 Ma (95% HPD: 34.72-45.36 Ma). The diversification of sect. *Cephalophilon* and of sect. *Echinocaulon* was estimated to have started at ca. 14.29 Ma (95% HPD: 11.11-16.68 Ma) and 17.39 Ma (95% HPD: 12.06-19.37 Ma), respectively, confidence interval overlapping with the Mid-Miocene Climatic Optimum (MMCO) (**Figure 4**).

## Discussion

### Chloroplast genome structure and comparative analysis

Our broad taxon sampling provides unprecedented insights into plastome structural evolution throughout Persicarieae (**Figure 1**). Plastome comparisons revealed high conservation within Persicarieae in structure and gene order. In this study, 74 plastomes were assembled, and all have typical quadripartite structures, as in most angiosperms, including LSC, SSC, and a pair of IRs, IRa and IRb (Palmer, 1985) (**Figure S1**). In *Bistorta*, *Koenigia* (except *K. delicatula*), and *P.* sect. *Cephalophilon*, the IR expanded to include *rps19*, *ndhF*, and *trnH*. Most *Koenigia* plastomes exhibit a conserved structure. However, *K. delicatula* lost the functional copies of all *ndh* genes. The loss of the NDH gene has been widely reported throughout plants and is often associated with unusual nutritional status (Funk et al., 2007; Wicke et al., 2013). We speculate that the dwarf and reddish leaf status of *K. delicatula* may be partly related to the loss of NDH (Qu et al., 2022). In Persicarieae, mononucleotide repeats predominated, followed by dinucleotide and trinucleotide repeats (**Table S4**). Most of the Persicarieae species SSRs were-composed solely of A or T as in plastomes of other Polygonaceae (Fan et al., 2021; Zhang et al., 2021). The plastomes of the Persicarieae species are rich in SSRs and could be good resources to develop SSRs markers for further studies.

### Comparison and evolution of morphological characters

**Figure 5:**
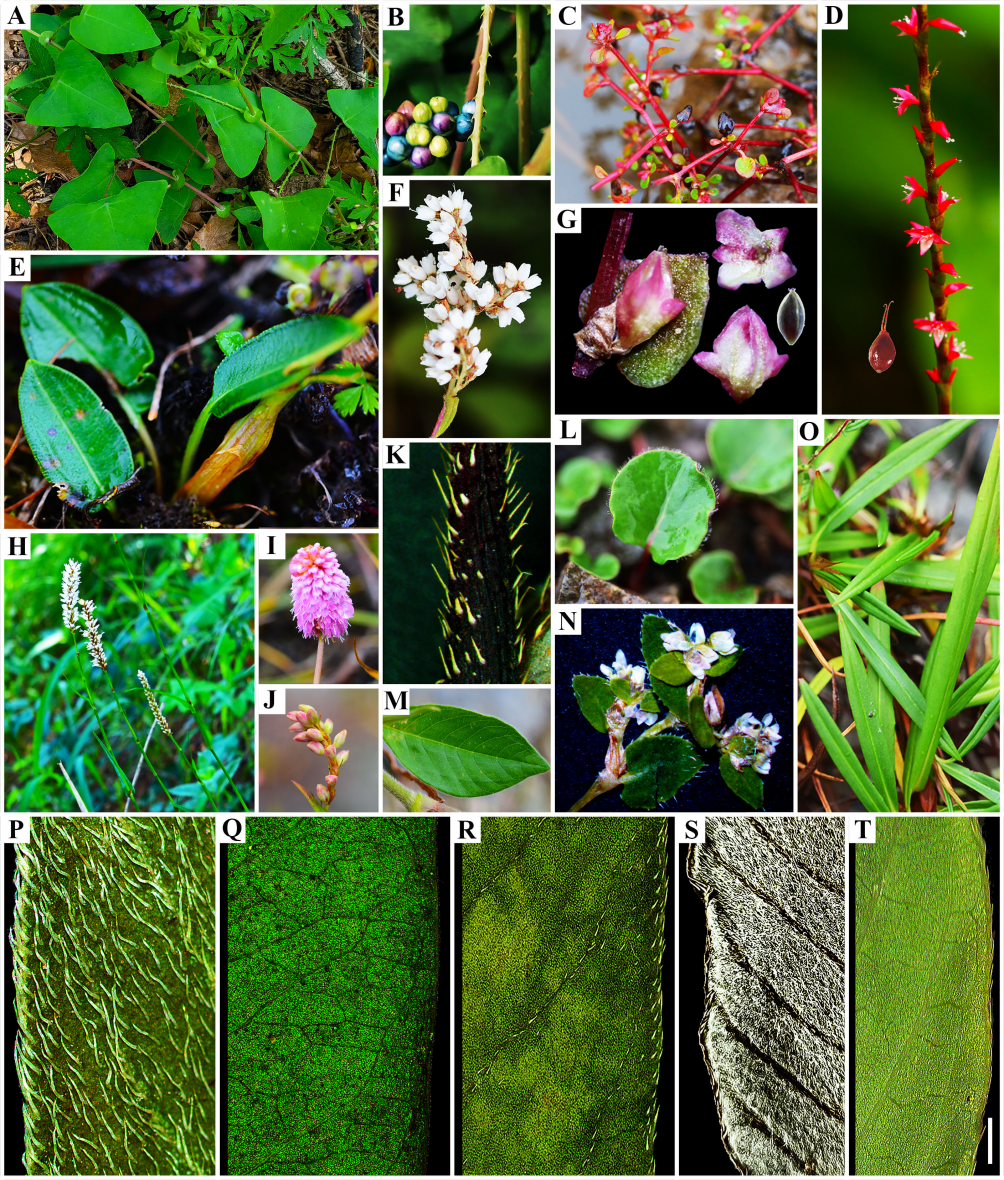
Morphological diversity in Persicarieae species. **(A)** and **(B)**, *Persicaria perfoliata*; **(C)**, *Koenigia islandica*; **(D)**, *P. filiformis*; **(E)** and **(I)**, *Bistorta macrophylla*; **(F)**, *K. lichiangensis*; **(G)**, *K. delicatula*; **(H)**, *B. officinalis*; **(J)** and **(O)**, *B. emodi*; **(K)** and **(R)**, *P. bungeana*; **(L)**, *K. forrestii*; **(M)**, *K. mollis var. rudis*; **(N)**, *K. cyanandra*; **(P)** and **(Q)**, *P. amphibia*; **(S)**, *P. lapathifolia var. salicifolia*; **(T)**, *P. lapathifolia*. Scale bar to all images. Scale bars: A, H = 30 mm; B, D, F, G, K, L, M–T = 5mm; C, J, E, I = 10 mm. Photo credits: Dong-Ling Cao and Jian Ru.

In the comparison of the systematic value of morphological characters in Persicarieae species, we recognized that they overlapped in morphospace. Because different characters are used as reference, the delineation of some species is different among taxonomists (Li et al., 2003; Min et al., 2013). Micromorphology is considered to be stable and significant, and researchers have examined subepidermal micromorphology (cell shape and stomatal type; Paul and Chowdhury, 2021), pollen morphology (Liang and Li, 2014; Kong et al., 2021), and fruit morphology (surface sculpture and pericarp micromorphology; Decraene and Smets, 2000; Chen and Zhou, 2012). The qualitative characters analyzed using PCA have been shown to have diagnostic importance among the taxa (Kong et al., 2021). The findings of the PCA indicated that these characters (including leaf venation, stomatal type, epidermal cell shape, inflorescence type, tepal venation, structure of exocarp slices, and fruit surface ornamentation) can separate Persicarieae, Fagopyreae, and Polygoneae (**Figure 2**). The results of PCA is consistent with the results of the phylogenetic tree, supporting Persicarieae as a natural unit. Studies showed that the venation of the tepals of all Persicarieae species is pinnate, rendering this a characteristic and stable trait of this taxon (Kong et al., 2018). In previous classifications, a three-merous perianth was considered ancestral (Sanchez et al., 2009), but the results of this study suggest that this is derived from a five-merous condition. Taxa with three- merous perianths only occur in *Koenigia*. Inflorescence type has long been an essential basis for determining the classification of subgroups in *Persicaria* (Li et al., 2003). Ancestral character state reconstruction suggests that the raceme may be the ancestral state from which capitate inflorescences may have evolved independently in *P.* sects. *Cephalophilon* and *Echinocaulon*, and paniculate inflorescences in *Koenigia* (**Figure 3C**). The number of styles changed only once, from three to two, in Persicarieae. Interestingly, within *Persicaria* there was a reversal from two to three styles in *P. viscosa* (**Figure 3D**). Our analysis of the flower evolution of Persicarieae supports 5-merous perianth, three styles, and eight stamens as the ancestral condition for the whole tribe. In *Persicaria*, stamen and style number of both sect. *Amphibia* and sect. *Tovara* tended to decrease, and the length of style tended to increase (**Figures 3D, E**; **5D**). Finally, pollen type was found to change multiple times during the evolution of Persicarieae. Some studies have shown that the pollen apertures of Polygonaceae evolve in a continuous direction (Zhou et al., 1999b; Zhou et al., 2000; Yasmin et al., 2010). To determine the evolutionary direction and the nature of the homogeneity of these characters, a more extensive and detailed morphological analysis is required.

### Phylogenetic relationships within *Persicaria*

Our results provide higher levels of resolution and support for relationships among the sections within *Persicaria* than in previous studies (**Figure 1**). At the section level, we first focus on the phylogenetic position of sect. *Cephalophilon* (**Figure 1E**). Species of sect. *Cephalophilon* are characterized by capitate inflorescences and 3-colpate pollen with coarse reticulate exine ornamentation. This section has been recognized as a natural group to be placed in *Persicaria* (Zhao et al., 2012; Kong and Hong, 2018; Kong et al., 2021). Sect. *Cephalophilon* is always at the base of the phylogenetic tree of nrITS and cpDNA, and it has always been considered the sister group to all other *Persicaria* sections (Zhao et al., 2012; Kong et al., 2021).

In previous phylogenetic studies of Persicarieae, strong conflicts between nrITS and cpDNA datasets have emerged. The main conflict centered on the branches involving *P. amphibia* and *P. filiformis*. Traditionally, *P. amphibia* has been placed in sect. *Persicaria* (Li et al., 2003). *Persicaria amphibia* is a highly polymorphic taxon that can grow in aquatic environments (*P. amphibia* 1 in **Figure 1**) as well as in moist terrestrial (*P. amphibia* 2 in **Figure 1**) habitats (Partridge, 2001; Kim and Donoghue, 2008b). Aquatic (**Figure 5Q**) and terrestrial (**Figure 5P**) plants of *P. amphibia* vary significantly in morphology. There is always a distinct split between *P. amphibia* and the remaining sections (Kim and Donoghue 2008a; Kim and Donoghue, 2008b; Schuster et al., 2011b). The split is also supported by morphological differences, such as the pollen apertures (Zhou et al., 1999a) and the anatomic structure of the leaf (Meng et al., 1997; Zhang, 2007). Therefore, some taxonomists, based on molecular data (ITS and cpDNA), proposed that this species merits its own section (Wang and Feng, 1994; Galasso et al., 2009; Qu and Xu, 2015). In this study, it is clear that *P. amphibia* 1 and *P. amphibia* 2 form a well-supported clade, whose isolated position is confirmed. Therefore, our study supports that the branch of *P. amphibia* should be treated as a separate section. A comparison of the pollen types of *Persicaria* (Yasmin et al., 2010; Kong et al., 2021) revealed that the outer wall sculptures of sect. *Persicaria*, *Amphibia*, and *Cephalophilon* were all coarsely reticulate, and their areoles all had granular or rod-like protrusions, indicating that these sections may be closely related. The clade where *P. filiformis* is located is different from other species of *Persicaria* in stigma number (2-hooked style) and perianth merosity (4-parted; **Figure 5D**), and there is an ongoing debate (Xu et al., 2011; Zhao et al., 2012; Min et al., 2013; Sun et al., 2014b) about the taxonomic rank. Some people recognize *Tovara* at generic rank based on this evidence, while others showed that these characters also occur in a number of taxa in section *Persicaria*. Molecular evidence (Youngbae et al., 1997; Xu et al., 2011) does not support the establishment of a separate genus, and results indicated the clade should be treated as section *Tovara*, which is consistent with previous analyses based on morphology (Decraene and Akeroyd, 1998; Decraene and Smets, 2000; Kong et al., 2021) and molecular data (Youngbae et al., 1997; Kim and Donoghue, 2008b; Xu et al., 2011; Schuster et al., 2015). In summary, there are five highly supported monophyletic groups (**Figure 1**) we suggest to classify as five sections, namely, *Persicaria*, *Amphibia*, *Tovara*, *Echinocaulon*, and *Cephalophilon* (**Figure 1**).

The morphological characters of *P. lapathifolia* (**Figure 5T**) and *P. lapathifolia* var. *salicifolia* (**Figure 5S**) are almost identical; the latter’s leaves differ in the presence of cottony hairs (filiform unicellular non-glandular hairs) on the abaxial epidermis. Studies have shown that the cottony hairs on the leaves of this species are the result of adaptation to environmental changes (Qu et al., 2006; Gang and Cai, 2008), and such hairy morphotypes should not be regarded as a variety of *P. lapathifolia*. In the present study, the sister relationship between the two varieties was well supported in both the species tree and the phylogenetic tree. Final assessment of the taxonomic status of the two varieties will require a broad geographical sampling beyond the scope of this study. *Persicaria bungeana* (**Figures 5K, R**) has long been placed in sect. *Echinocaulon* (**Figures 5A, B**) because of the presence of typical retrorse prickles on the stem. Our dataset set strongly supports (Dong, 2012; Min et al., 2013) the transfer of *P. bungeana* to sect. *Persicaria* (**Figure 1**). Morphologically, *P. bungeana*, which has a raceme as inflorescence and simple leaves, is similar to other species of section *Persicaria*.

### Phylogenetic relationships of *Koenigia* and *Bistorta*

*Aconogonon* and *Koenigia* share similar distributional areas and numerous characters, including trichome type, inflorescence, and tepal epidermal cells (**Table S2**). This demonstrated the difficulties in distinguishing between *Aconogonon* and *Koenigia* based only on morphological traits. Here, we present robust results based on expanded taxon sampling with careful morphological comparisons (**Figures 1**, **5**). Based on our results and previous analyses (Galasso et al., 2009; Fan et al., 2013; Schuster et al., 2015), we support merging *Aconogonon* and *Koenigia*. This treatment solves the current problem of nesting and makes the taxon natural (**Figures 1F, G**). Therefore, the long-disputed phylogenetic position of *K. forrestii* (**Figure 5L**) is resolved. The results of this study support *K. cyanandra* (**Figure 5N**) as a member of *Koenigia*, further resolving the contradiction of previous studies based on molecular fragments and morphology (Liu et al., 2007; Zhao et al., 2012). The previous classification (Min, 2013) of *Koenigia* into three sections based on fruit micromorphology was not supported in the present study.

In *Bistorta*, both inadequate informative sites of DNA sequences and a limited number of taxon sampling have sometimes led to poor phylogenetic resolution and inappropriate taxonomic treatment (**Figures 5E, H, I**). The classification of *Bistorta* into three sections based on leaf venation in previous studies (Liu et al., 2006) is partly supported here (*B. vaccinifolia* was not collected). Notably, *B. emodi* is the only small shrub within this clade with a pollen type and leaf venation type distinctly different from other species of the clade (**Figures 5O, J**). Therefore, we preliminarily support that sect. *Emodi* should be placed in *Bistorta* (Liu et al., 2006). Further analyses are still required to obtain a suitable interpretation of this phenomenon and to resolve the phylogenetic relationships among *Bistorta* species, based on the sampling of as many taxa as possible.

### Evolutionary patterns in Persicarieae

Recent studies on spatial and temporal differences in plant diversity point to southern China as the center of diversity for both ancient and young genera (Hu et al., 2021). According to biogeographic analysis of Polygonaceae (Zhou et al., 1999a; Wang et al., 2007), southwest China is the origin and diversification center of Persicarieae. Since southwest China is adjacent to the Tibetan Plateau, it constitutes the southeastern edge of the Tibetan Plateau, a region with unusually complex and diverse geomorphological and climatic features (Sun et al., 2017; Xia et al., 2022). Incredibly high inter- and intra-specific diversification rates of plants have been documented in this region, hypothetically attributed to the geological uplifts of the QTP (Baldwin, 2014; Mao et al., 2021; Xu and Chen, 2021). Studies have demonstrated that climatic oscillations in the region have triggered the radiation/diversification of plants in several genera (Zhang et al., 2014a; He et al., 2020). Estimates of divergence time indicate that Persicarieae diverged approximately 55.8 Ma, which is close to the Paleocene-Eocene Thermal Maximum (PETM) with global warming in general (**Figure 4**). Radiation of *Koenigia* and *Persicaria* began shortly thereafter. As fast evolution is an adaptation strategy for climate change, these significant geological fluctuations may have led to the divergence of Persicarieae species. After the west wind passes through the southern part of the QTP, it gradually warms up, and turns into a southwesterly airflow with a higher humidity, resulting in a moist climate in the southwest (Schiemann et al., 2009; Zhu et al., 2015). In addition, the sea-land monsoon forms a north-south water vapor channel that also has an important impact on the warm and humid climate of the southwest such as higher mean annual temperature and greater mean annual rainfall (Li et al., 2014; Hui et al., 2018). The diversification time of *P.* sects. *Echinocaulon* and *Cephalophilon* is estimated to be 14.29 Ma (95% HPD: 11.11-16.68 Ma) and 17.39 Ma (95% HPD: 12.06-19.37 Ma), which is near to the Middle Miocene Climatic Optimum (MMCO) (**Figure 4**). The study showed that the sporopollen content of hygrophyte continued to increase throughout the MMCO period (Xie et al., 2017; Hui et al., 2018). The role of increased temperature as a driver of biodiversity was previously acknowledged (Julie et al., 2016; Hui et al., 2018), and it is thought to reflect increased rates of biological processes and the consequent higher speciation rates (Kong et al., 2017). We suggest that the diversity of vegetation and environments, as well as the overall warm and humid climate of the Miocene, facilitated the divergent evolution of *Persicaria* species. This further explains why *Persicaria* species are usually better adapted and thrive in wet environments in the wild.

The *Koenigia* species, distributed mainly in the Hengduan Mountains (HDM) and the Himalayas (Li et al., 2003), originated approximately 48.66 Ma (95% HPD: 47.24-49.79 Ma) (**Figure 4**). *Koenigia delicatula* diverged from other *Koenigia* species ca. 44.80 Ma (95% HPD: 43.27-46.60 Ma), followed by a period of low global temperatures and an arid climate. After about 37 Ma, humidity and temperature increased again, and other species of *Koenigia* successively differentiated (**Figure 4**). We noted that *Koenigia* species have small and thick leaves, small epidermal cell size, and high stomatal density and stomatal index, which are all characters of their adaptation to intense light at high altitudes (**Figures 5C, F, G, M**). More importantly, the small and dense stomata help to prevent their water loss due to transpiration (Kannenberg et al., 2018). Studies have shown that the various forms of succulence are the result of specialized ecological adaptations evolving in concert with these extreme environments (Yao et al., 2019). We inferred that *Koenigia* is a young floristic element formed on the Tibetan plateau rising during the Himalayan orogeny. It is noteworthy that most perennial clades in *Koenigia* are young, e.g., *K. lichiangensis* originated 0.5 Ma (**Figure 5F**). Perennial plants in alpine settings are better able to use water than annual plants and play a vital role in preserving the functioning of the community (Li et al., 2007). Studies have shown that alpine ecosystems existed in parts of the QTP during the Oligocene, and since the Late Miocene, the landscape and climate patterns of different components of the plateau have changed significantly, with ancestral taxa of alpine organisms moving into the uplifting plateau or evolving new species adapted to alpine habitats in place on the plateau (Mao et al., 2021). Intriguingly, *B. emodi* diverged around 26 Ma during the Late Oligocene, while the other species of the clade diverged around 5 Ma during the early Pliocene. The large difference in paleoclimatic environments between these two periods could further explain the morphological differences between *B. emodi* and other species (**Figure 4**). Biogeographic studies have found that the alpine regions of the Tibetan Plateau and surrounding areas are closely linked to the high latitudes of the Northern Hemisphere. A large ice cap covering the entire Tibetan Plateau did not form during the Quaternary Ice Age, and there are still many biological refuges on the plateau surface (Liu et al., 2014). Core-*Bistorta* diversification occurred between the end of the Neogene and the beginning of the Quaternary (**Figure 4**). We found that the evolution trend of the *Bistorta* leaves was that the size became smaller, and the basal leaves of young groups were generally narrow linear or lanceolate. Some studies (Farber et al., 2016; Gerlein-Safdi et al., 2018) have shown that smaller leaves can reduce transpiration and heat loss, which is beneficial for plants to adapt to low temperature environment.

## Conclusion

This study has advanced our knowledge of Persicarieae phylogeny. We presented a comparative analysis of 80 plastomes and reported a comprehensive study of morphology, phylogenetic relationships, divergence time estimation, and ancestral state reconstruction. Highly diversified morphological characters of Persicarieae likely adaptively evolved to cope with various habitats. This study provides reference information on studying the timing and drivers of species divergence on the Tibetan Plateau and adjacent areas.

## Data availability statement

The datasets presented in this study can be found in online repositories. The names of the repository/repositories and accession number(s) can be found in the article/ **Supplementary Material**.

## Author contributions

S-JF, X-JQ, and D-LC participated in the conception and design of the research framework. S-JF and X-JZ collected and identified the species. D-LC was responsible for analyzing and processing data and writing the manuscript. S-JF, X-JQ, and X-JZ revised the manuscript. All authors contributed to the article and approved the submitted version.

## Supplementary material

Supplementary Figure 1 Comparisons of LSC, SSC, and IR region borders among Persicarieae species. The IR regions have the same color. Color coding indicates different genes on both sides of the junctions. The number above the gene features means the distance between the ends of genes and the junction sites.

Supplementary Figure 2 Sequence alignment of Persicarieae plastomes using mVISTA. The position and transcriptional direction of each gene are indicated by gray arrows. The vertical scale indicates the percentage of identity, ranging from 50 to 100%. The horizontal axis indicates the coordinates within the plastome. Genome regions are color-coded as exons, introns, and intergenic spacer (IGS).

Supplementary Figure 3 The nucleotide diversity (Pi) values of the Persicarieae plastomes; Pi values of coding genes and Pi values of IGS. The red arrows point to the 12 most highly variable loci.

Supplementary Figure 4 ML phylogenetic tree inferred from 80 individuals based on 79 PCGs using RAxML. Numbers next to each node denote bootstrap support values.

Supplementary Figure 5 ML phylogenetic tree of 80 individuals based on noncoding plastome sequences.

Supplementary Figure 6 Species tree inferred from 79 PCGs using ASTRAL. Numbers next to each node are the posterior probability values.
