# Supplementary figures and images for: Plastid phylogenomics sheds light on divergence time and ecological adaptations of the tribe Persicarieae (Polygonaceae)
Source: Front Plant Sci. 2022 Dec 8;13:1046253. doi: 10.3389/fpls.2022.1046253 (PMC9780030; doi:10.3389/fpls.2022.1046253)

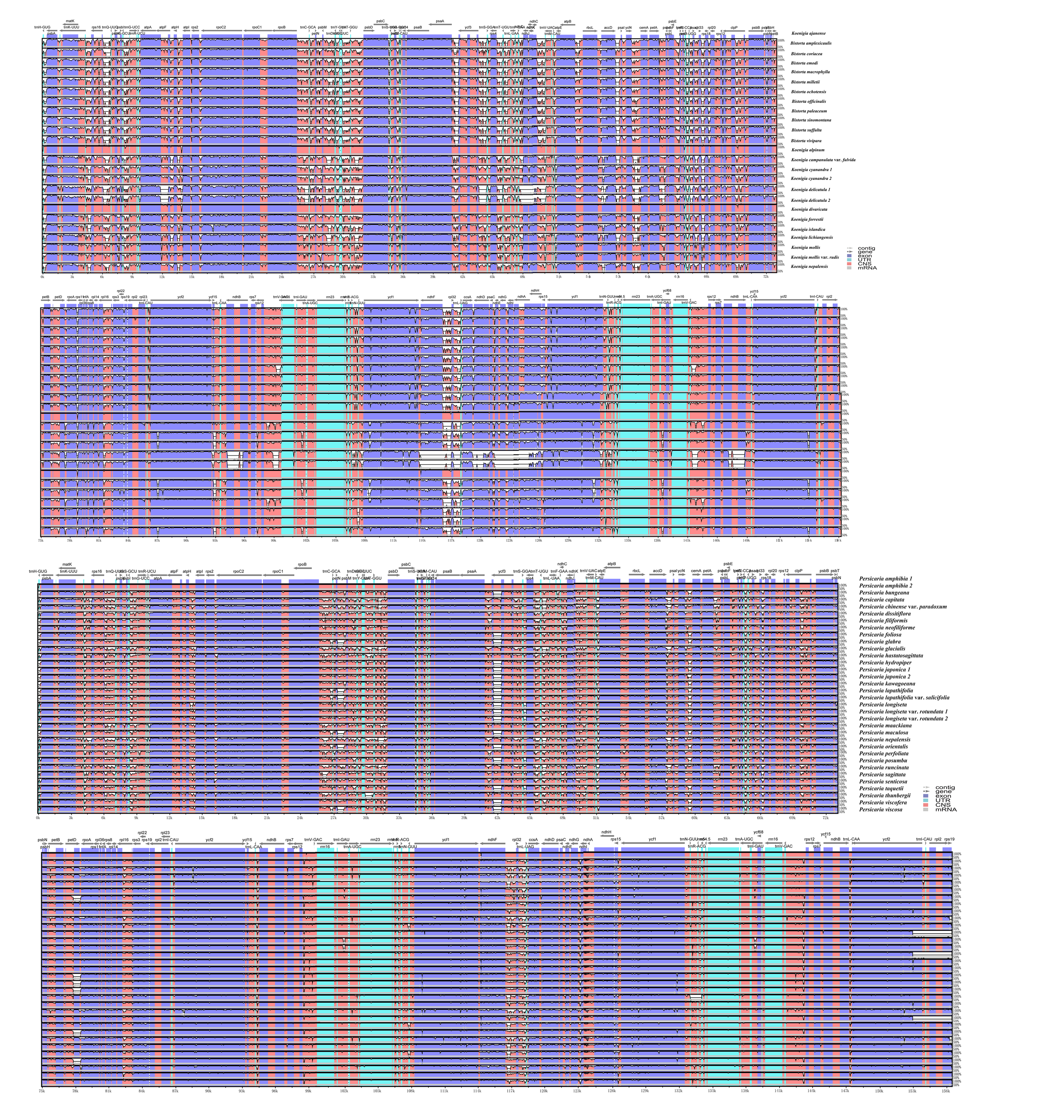

Supplement: Supplementary Figure 1 — Comparisons of LSC, SSC, and IR region borders among Persicarieae species. The IR regions have the same color. Color coding indicates different genes on both sides of the junctions. The number above the gene features means the distance between the ends of genes and the junction sites. [file DataSheet_1.zip › Image 2.JPEG]

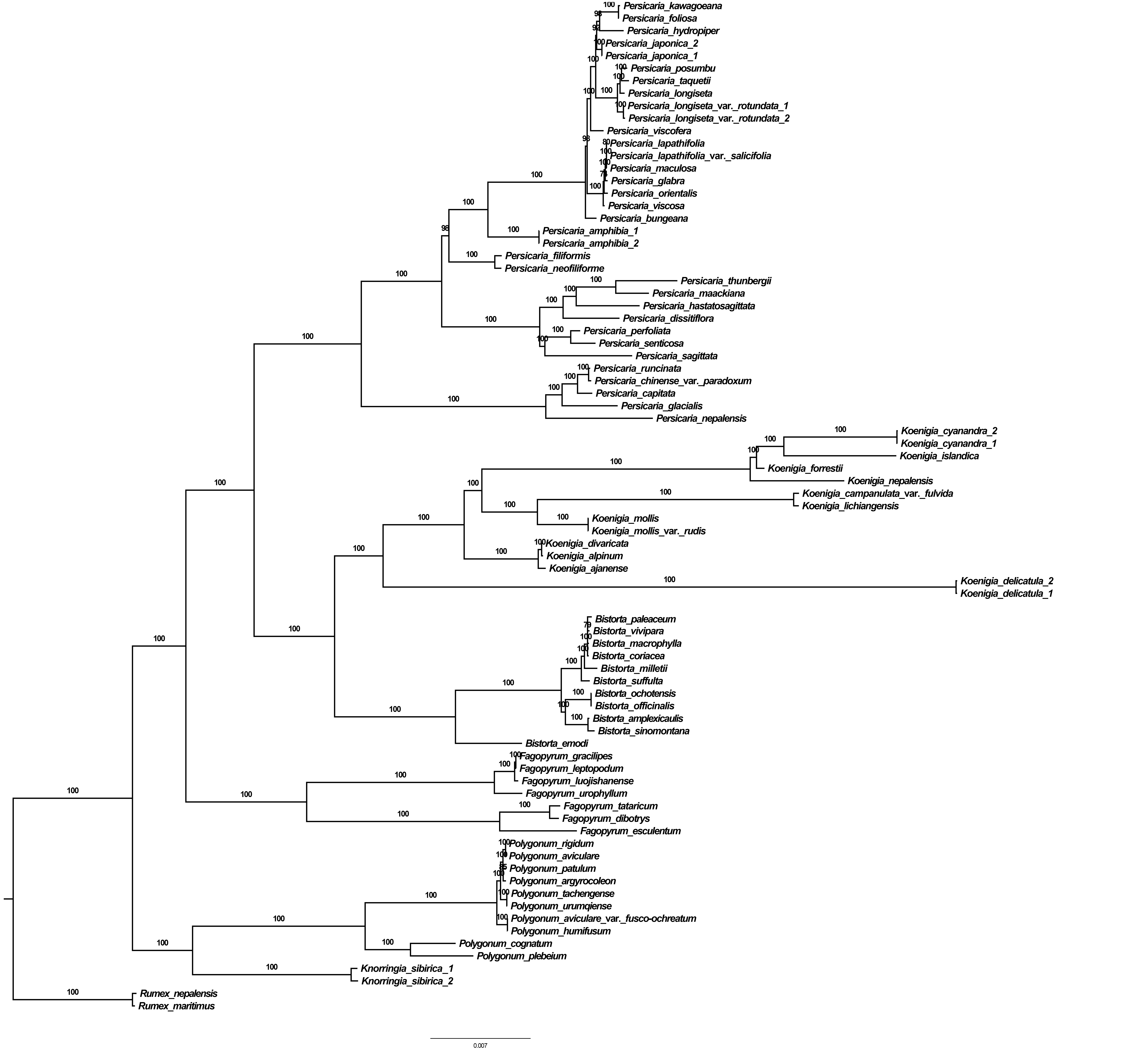

Supplement: Supplementary Figure 1 — Comparisons of LSC, SSC, and IR region borders among Persicarieae species. The IR regions have the same color. Color coding indicates different genes on both sides of the junctions. The number above the gene features means the distance between the ends of genes and the junction sites. [file DataSheet_1.zip › Image 4.JPEG]

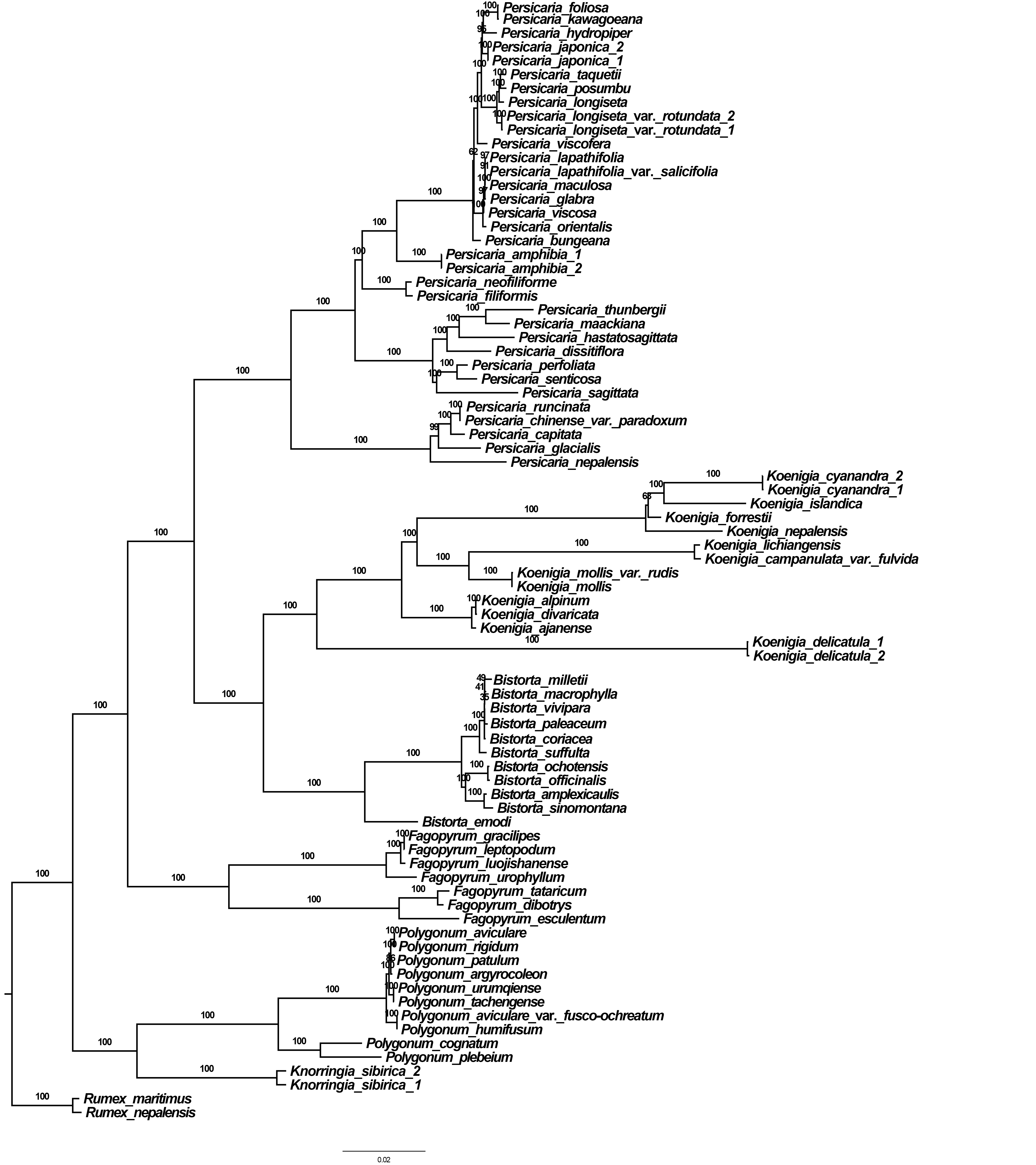

Supplement: Supplementary Figure 1 — Comparisons of LSC, SSC, and IR region borders among Persicarieae species. The IR regions have the same color. Color coding indicates different genes on both sides of the junctions. The number above the gene features means the distance between the ends of genes and the junction sites. [file DataSheet_1.zip › Image 5.JPEG]

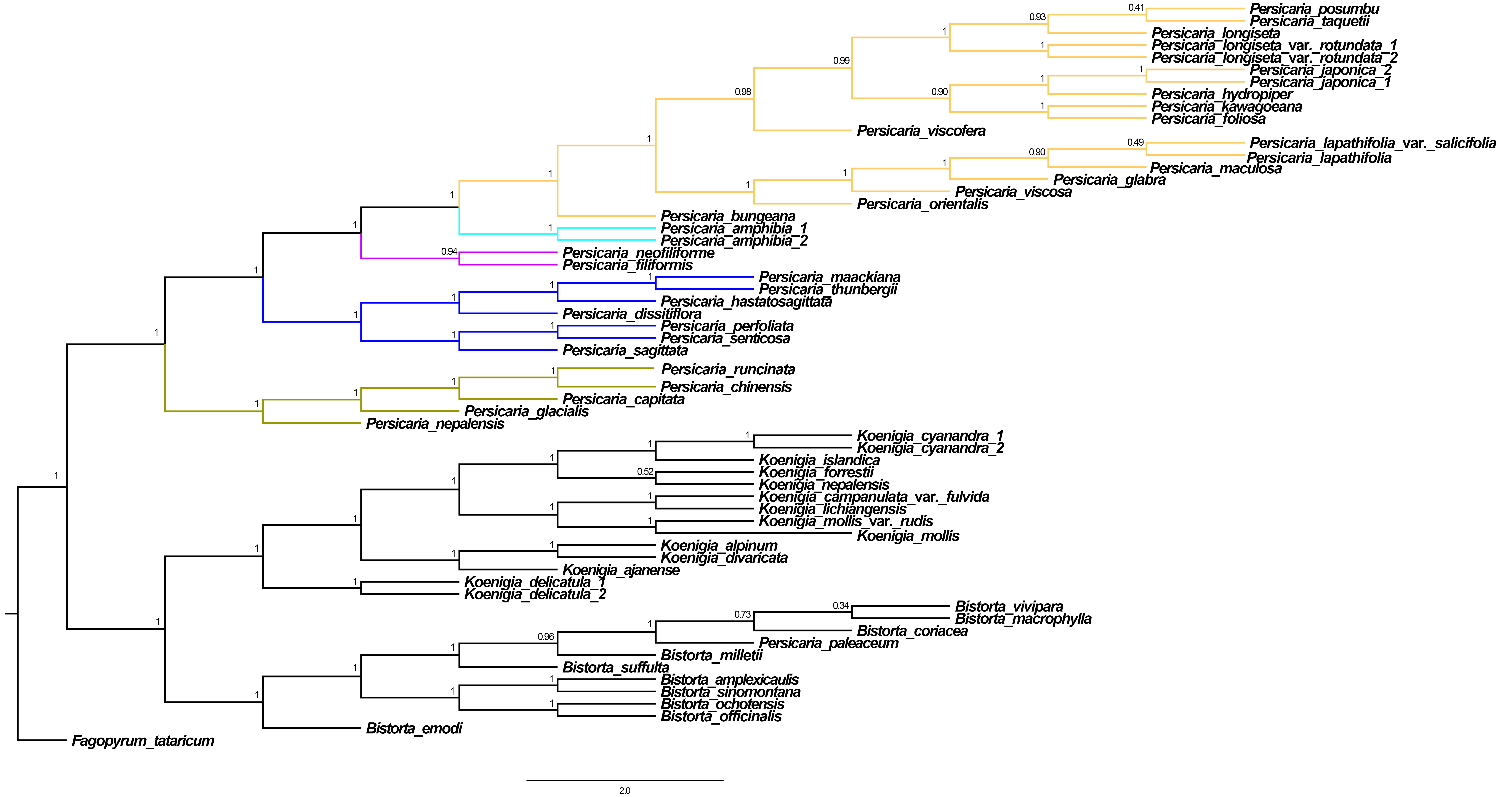

Supplement: Supplementary Figure 1 — Comparisons of LSC, SSC, and IR region borders among Persicarieae species. The IR regions have the same color. Color coding indicates different genes on both sides of the junctions. The number above the gene features means the distance between the ends of genes and the junction sites. [file DataSheet_1.zip › Image 6.JPEG]
