# Supplementary material for: Plastid phylogenomics sheds light on divergence time and ecological adaptations of the tribe Persicarieae (Polygonaceae)
Source: Front Plant Sci. 2022 Dec 8;13:1046253. doi: 10.3389/fpls.2022.1046253 (PMC9780030; doi:10.3389/fpls.2022.1046253)
Supplement: Supplementary file 2 [file DataSheet_2.zip › Table 2.DOCX]

**Table S2** Details of the morphological traits used for PCA analysis.

| **Trait/Type** | **I** | **II** | **III** | **IV** | **V** | **VI** | **VII** | **VIII** | **IX** | **X** |
| --- | --- | --- | --- | --- | --- | --- | --- | --- | --- | --- |
| **Leaf venation** | pinnate venation | palmate venation | - | - | - | - | - | - | - | - |
| **Tepal venation** | pinnate venation | 3-basinerved | - | - | - | - | - | - | - | - |
| **Leaf abaxial epidermal cell shape** | polygonal | irregular | - | - | - | - | - | - | - | - |
| **Stomata type** | anomocytic | anisocytic | paracytic | mixture of both | - | - | - | - | - | - |
| **Fruit surface**  **sculpture** | neoplastic | smooth | warty | pits | reticulation | brain stripe | folds | mound | stellate | clutter |
| **Exocarp structure** | absent | dichotomous | dichotomous, trichotomous,  dendritic, or absent | - | - | - | - | - | - | - |
| **Inflorescence** | raceme | panicle | capitulum | cyme | solitary | corymb | - | - | - | - |

| **Species/Trait** | **Leaf venation** | **Tepal venation** | **Leaf abaxial epidermal cell shape** | **Stomata type** | **Fruit surface sculpture** | **Exocarp structure**  **(branch)** | **Inflorescence** |
| --- | --- | --- | --- | --- | --- | --- | --- |
| ***Polygonum rigidum*** | pinnate venation | pinnate venation | polygonal | anisocytic | neoplastic | dichotomous, trichotomous,  dendritic, or absent | solitary |
| ***Polygonum aviculare*** | pinnate venation | pinnate venation | polygonal | anisocytic | neoplastic | dichotomous, trichotomous,  dendritic, or absent | solitary |
| ***Polygonum argyrocoleon*** | pinnate venation | pinnate venation | polygonal | anisocytic | smooth | dichotomous, trichotomous,  dendritic, or absent | solitary |
| ***Polygonum patulum*** | pinnate venation | pinnate venation | polygonal | anisocytic | neoplastic | dichotomous, trichotomous,  dendritic, or absent | solitary |
| ***Polygonum tachengense*** | pinnate venation | pinnate venation | polygonal | anisocytic | warty | dichotomous, trichotomous,  dendritic, or absent | solitary |
| ***Polygonum urumqiense*** | pinnate venation | pinnate venation | irregular | anisocytic | neoplastic | dichotomous, trichotomous,  dendritic, or absent | solitary |
| ***Polygonum humifusum*** | pinnate venation | pinnate venation | polygonal | anisocytic | neoplastic | dichotomous, trichotomous,  dendritic, or absent | solitary |
| ***Polygonum aviculare var. fusco-ochreatum*** | pinnate venation | pinnate venation | polygonal | anisocytic | neoplastic | dichotomous, trichotomous,  dendritic, or absent | solitary |
| ***Polygonum plebeium*** | pinnate venation | pinnate venation | polygonal | anisocytic | smooth | dichotomous, trichotomous,  dendritic, or absent | solitary |
| ***Polygonum cognatum*** | pinnate venation | pinnate venation | polygonal | anisocytic | smooth | dichotomous, trichotomous,  dendritic, or absent | solitary |
| ***Knorringia sibirica*** | pinnate venation | pinnate venation | polygonal | anisocytic | pits | dichotomous | panicle |
| ***Fagopyrum leptopodum*** | palmate venation | pinnate venation | irregular | mixture of both | neoplastic | absent | raceme |
| ***Fagopyrum gracilipes*** | palmate venation | pinnate venation | irregular | mixture of both | neoplastic | absent | raceme |
| ***Fagopyrum urophyllum*** | palmate venation | pinnate venation | irregular | mixture of both | neoplastic | absent | raceme |
| ***Fagopyrum dibotrys*** | palmate venation | 3-basinerved | irregular | mixture of both | reticulation | absent | corymb |
| ***Fagopyrum tataricum*** | palmate venation | 3-basinerved | irregular | mixture of both | reticulation | absent | raceme |
| ***Fagopyrum esculentum*** | palmate venation | 3-basinerved | irregular | mixture of both | reticulation | absent | corymb |
| ***Persicaria taquetii*** | pinnate venation | 3-basinerved | irregular | paracytic | reticulation | dichotomous | raceme |
| ***Persicaria posumbu*** | pinnate venation | 3-basinerved | irregular | paracytic | brain stripe | dichotomous | raceme |
| ***Persicaria longiseta*** | pinnate venation | 3-basinerved | irregular | paracytic | brain stripe | dichotomous | raceme |
| ***Persicaria longiseta* var. *rotundata*** | pinnate venation | 3-basinerved | irregular | paracytic | reticulation | dichotomous | raceme |
| ***Persicaria foliosa*** | pinnate venation | 3-basinerved | irregular | paracytic | pits | dichotomous | raceme |
| ***Persicaria kawagoeana*** | pinnate venation | 3-basinerved | irregular | paracytic | reticulation | dichotomous | raceme |
| ***Persicaria hydropiper*** | pinnate venation | 3-basinerved | irregular | paracytic | reticulation | dichotomous | raceme |
| ***Persicaria japonica*** | pinnate venation | 3-basinerved | irregular | paracytic | brain stripe | dichotomous | raceme |
| ***Persicaria viscofera*** | pinnate venation | 3-basinerved | irregular | anisocytic | pits | dichotomous | raceme |
| ***Persicaria lapathifolia* var*. salicifolia*** | pinnate venation | 3-basinerved | irregular | anisocytic | pits | dichotomous | raceme |
| ***Persicaria lapathifolia*** | pinnate venation | 3-basinerved | irregular | anisocytic | pits | dichotomous | raceme |
| ***Persicaria maculosa*** | pinnate venation | 3-basinerved | irregular | anisocytic | brain stripe | dichotomous | raceme |
| ***Persicaria glabra*** | pinnate venation | 3-basinerved | irregular | paracytic | folds | dichotomous | raceme |
| ***Persicaria orientalis*** | pinnate venation | 3-basinerved | irregular | mixture of both | brain stripe | dichotomous | raceme |
| ***Persicaria viscosa*** | pinnate venation | 3-basinerved | irregular | anisocytic | warty | dichotomous | raceme |
| ***Persicaria bungeana*** | pinnate venation | 3-basinerved | irregular | anisocytic | warty | dichotomous | raceme |
| ***Persicaria amphibia*** | pinnate venation | 3-basinerved | polygonal | anisocytic | pits | dichotomous | raceme |
| ***Persicaria neofiliformis*** | pinnate venation | 3-basinerved | irregular | anisocytic | pits | absent | raceme |
| ***Persicaria filiformis*** | pinnate venation | 3-basinerved | irregular | anisocytic | pits | absent | raceme |
| ***Persicaria maackiana*** | palmate venation | 3-basinerved | irregular | paracytic | folds | absent | raceme |
| ***Persicaria thunbergii*** | palmate venation | 3-basinerved | irregular | paracytic | folds | absent | raceme |
| ***Persicaria hastatosagittata*** | palmate venation | 3-basinerved | irregular | paracytic | folds | absent | raceme |
| ***Persicaria dissitiflora*** | palmate venation | 3-basinerved | irregular | paracytic | / | absent | panicle |
| ***Persicaria perfoliata*** | palmate venation | 3-basinerved | irregular | paracytic | pits | absent | raceme |
| ***Persicaria senticosa*** | palmate venation | 3-basinerved | irregular | paracytic | mound | absent | capitulum |
| ***Persicaria sagittata*** | palmate venation | 3-basinerved | irregular | paracytic | neoplastic | absent | capitulum |
| ***Persicaria chinense* var*. paradoxum*** | pinnate venation | 3-basinerved | irregular | mixture of both | reticulation | absent | capitulum |
| ***Persicaria runcinata*** | pinnate venation | 3-basinerved | irregular | mixture of both | stellate | absent | capitulum |
| ***Persicaria capitata*** | pinnate venation | 3-basinerved | irregular | mixture of both | folds | absent | capitulum |
| ***Persicaria glacialis*** | pinnate venation | 3-basinerved | irregular | anomocytic | reticulation | absent | capitulum |
| ***Persicaria nepalensis*** | pinnate venation | 3-basinerved | irregular | anomocytic | neoplastic | absent | capitulum |
| ***Koenigia cyanandra*** | pinnate venation | 3-basinerved | irregular | anomocytic | warty | dichotomous | capitulum |
| ***Koenigia islandica*** | pinnate venation | 3-basinerved | irregular | mixture of both | stellate | absent | capitulum |
| ***Koenigia forrestii*** | pinnate venation | 3-basinerved | irregular | mixture of both | stellate | dichotomous | cyme |
| ***Koenigia nepalensis*** | pinnate venation | 3-basinerved | irregular | mixture of both | brain stripe | dichotomous | capitulum |
| ***Koenigia campanulata* var*. fulvida*** | pinnate venation | 3-basinerved | irregular | mixture of both | brain stripe | dichotomous | panicle |
| ***Koenigia lichiangensis*** | pinnate venation | 3-basinerved | irregular | mixture of both | stellate | dichotomous | panicle |
| ***Koenigia mollis* var. *rudis*** | pinnate venation | 3-basinerved | irregular | mixture of both | stellate | dichotomous | panicle |
| ***Koenigia mollis*** | pinnate venation | 3-basinerved | irregular | mixture of both | stellate | dichotomous | panicle |
| ***Koenigia divaricata*** | pinnate venation | 3-basinerved | irregular | mixture of both | stellate | dichotomous | panicle |
| ***Koenigia alpinum*** | pinnate venation | 3-basinerved | irregular | mixture of both | clutter | dichotomous | panicle |
| ***Koenigia ajanense*** | pinnate venation | 3-basinerved | irregular | mixture of both | clutter | dichotomous | panicle |
| ***Koenigia delicatula*** | pinnate venation | 3-basinerved | irregular | anomocytic | stellate | dichotomous | capitulum |
| ***Bistorta paleaceum*** | pinnate venation | 3-basinerved | irregular | paracytic | stellate | dichotomous | raceme |
| ***Bistorta vivipara*** | pinnate venation | 3-basinerved | irregular | mixture of both | stellate | dichotomous | raceme |
| ***Bistorta macrophylla*** | pinnate venation | 3-basinerved | irregular | mixture of both | stellate | dichotomous | raceme |
| ***Bistorta milletii*** | pinnate venation | 3-basinerved | irregular | **/** | stellate | dichotomous | raceme |
| ***Bistorta suffulta*** | pinnate venation | 3-basinerved | irregular | mixture of both | stellate | dichotomous | raceme |
| ***Bistorta officinalis*** | pinnate venation | 3-basinerved | irregular | anisocytic | stellate | dichotomous | raceme |
| ***Bistorta ochotensis*** | pinnate venation | 3-basinerved | irregular | mixture of both | stellate | dichotomous | raceme |
| ***Bistorta amplexicaulis*** | pinnate venation | 3-basinerved | irregular | mixture of both | stellate | dichotomous | raceme |
| ***Bistorta sinomontana*** | pinnate venation | 3-basinerved | irregular | anisocytic | stellate | dichotomous | raceme |
| ***Bistorta emodi*** | pinnate venation | 3-basinerved | irregular | paracytic | pits | dichotomous | raceme |

Note: “/” means there is no relevant information.
