# Supplementary material for: Plastid phylogenomics sheds light on divergence time and ecological adaptations of the tribe Persicarieae (Polygonaceae)
Source: Front Plant Sci. 2022 Dec 8;13:1046253. doi: 10.3389/fpls.2022.1046253 (PMC9780030; doi:10.3389/fpls.2022.1046253)
Supplement: Supplementary file 2 [file DataSheet_2.zip › Table 3.DOCX]

**Table S3** Plastome characteristics of 59 Persicarieae individuals.

| **Species** | **Genome Size (bp)** | **LSC region (bp)** | **IR region (bp)** | **SSC region (bp)** | **GC content (%)** | | | |
| --- | --- | --- | --- | --- | --- | --- | --- | --- |
|  |  |  |  |  | **Over all** | **LSC** | **IR** | **SSC** |
| *Bistorta amplexcaulis* | 159789 | 84220 | 31072 | 13425 | 37.9 | 36.1 | 41.4 | 32.6 |
| *Bistorta coriacea* | 158839 | 83783 | 30947 | 13162 | 38.0 | 36.3 | 41.4 | 32.9 |
| *Bistorta emodi* | 161117 | 85853 | 31074 | 13116 | 37.6 | 35.7 | 41.4 | 32.6 |
| *Bistorta macrophylla* | 158838 | 83773 | 30948 | 13169 | 38.0 | 36.3 | 41.4 | 32.9 |
| *Bistorta milletii* | 159068 | 83771 | 31070 | 13157 | 38.0 | 36.3 | 41.3 | 32.8 |
| *Bistorta ochotensis* | 159626 | 84114 | 31069 | 13374 | 37.9 | 36.1 | 41.4 | 32.7 |
| *Bistorta officinalis* | 159714 | 84092 | 31096 | 13430 | 37.8 | 36.1 | 41.4 | 32.6 |
| *Bistorta paleaceum* | 158866 | 83780 | 30951 | 13184 | 38.0 | 36.3 | 41.4 | 32.9 |
| *Bistorta sinomontana* | 159075 | 84198 | 30712 | 13453 | 37.9 | 36.1 | 41.4 | 32.5 |
| *Bistorta suffulta* | 159115 | 84075 | 30934 | 13172 | 38.0 | 36.2 | 41.4 | 32.9 |
| *Bistorta vivipara* | 158852 | 83797 | 30947 | 13161 | 38.0 | 36.3 | 41.4 | 32.9 |
| *Koenigia ajanense* | 159220 | 83716 | 31060 | 13384 | 37.4 | 35.5 | 41.2 | 31.9 |
| *Koenigia alpinum* | 159135 | 83638 | 31058 | 13381 | 37.4 | 35.5 | 41.2 | 31.9 |
| *Koenigia campanulata* var. *fulvida* | 154446 | 80445 | 30798 | 12405 | 37.9 | 36.2 | 41.2 | 32.6 |
| *Koenigia cyanandra* 1 | 155971 | 81856 | 30585 | 12945 | 37.2 | 35.2 | 41.1 | 31.1 |
| *Koenigia cyanandra* 2 | 155973 | 81856 | 30586 | 12945 | 37.2 | 35.2 | 41.1 | 31.0 |
| *Koenigia delicatula* 1 | 138394 | 77434 | 28403 | 4154 | 37.9 | 35.7 | 41.3 | 30.6 |
| *Koenigia delicatula* 2 | 138438 | 77478 | 28403 | 4154 | 37.8 | 35.7 | 41.3 | 30.5 |
| *Koenigia divaricata* | 159126 | 83630 | 31058 | 13380 | 37.4 | 35.5 | 41.2 | 32.0 |
| *Koenigia forrestii* | 156798 | 82547 | 30590 | 13071 | 37.3 | 35.3 | 41.1 | 31.6 |
| *Koenigia islandica* | 155854 | 81593 | 30588 | 13085 | 37.2 | 35.2 | 41.1 | 31.1 |
| *Koenigia lichiangensis* | 154524 | 80498 | 30806 | 12414 | 37.9 | 36.1 | 41.2 | 32.7 |
| *Koenigia mollis* | 158578 | 83658 | 31107 | 12706 | 37.5 | 35.5 | 41.2 | 32.3 |
| *Koenigia mollis* var. *rudis* | 158627 | 83688 | 31107 | 12725 | 37.5 | 35.5 | 41.2 | 32.3 |
| *Koenigia nepalensis* | 155838 | 81616 | 30595 | 13032 | 37.4 | 35.5 | 41.2 | 31.4 |
| *Persicaria amphibia* 1 | 159464 | 84290 | 30956 | 13262 | 38.2 | 36.6 | 41.4 | 32.8 |
| *Persicaria amphibia* 2 | 159464 | 84290 | 30956 | 13262 | 38.2 | 36.6 | 41.4 | 32.8 |
| *Persicaria bungeana* | 159660 | 84205 | 31140 | 13175 | 38.2 | 36.5 | 41.4 | 33.2 |
| *Persicaria capitata* | 158852 | 84090 | 30931 | 12900 | 38.0 | 36.1 | 41.4 | 33.3 |
| *Persicaria chinense* var. *paradoxum* | 158981 | 84347 | 30872 | 12890 | 38.0 | 36.1 | 41.4 | 33.4 |
| *Persicaria dissitiflora* | 160982 | 85629 | 31175 | 13003 | 37.8 | 35.9 | 41.4 | 32.8 |
| *Persicaria filiformis* | 159721 | 84401 | 31123 | 13074 | 37.9 | 36.1 | 41.3 | 32.6 |
| *Persicaria foliosa* | 159693 | 84228 | 31135 | 13195 | 38.2 | 36.6 | 41.5 | 33.2 |
| *Persicaria glabra* | 159069 | 83636 | 31141 | 13151 | 38.2 | 36.6 | 41.5 | 33.2 |
| *Persicaria glacialis* | 158402 | 83661 | 30931 | 12879 | 38.0 | 36.1 | 41.4 | 33.3 |
| *Persicaria hastatosagittata* | 160798 | 85494 | 31130 | 13044 | 37.7 | 35.8 | 41.3 | 32.4 |
| *Persicaria hydropiper* | 159843 | 84350 | 31171 | 13151 | 38.2 | 36.5 | 41.5 | 33.2 |
| *Persicaria japonica* 1 | 159502 | 84342 | 30991 | 13178 | 38.2 | 36.6 | 41.4 | 33.1 |
| *Persicaria japonica* 2 | 159747 | 85013 | 30778 | 13178 | 38.1 | 36.5 | 41.5 | 33.1 |
| *Persicaria kawagoeana* | 159690 | 84241 | 31135 | 13179 | 38.2 | 36.6 | 41.5 | 33.2 |
| *Persicaria lapathifolia* | 159051 | 83620 | 31141 | 13149 | 38.2 | 36.6 | 41.5 | 33.2 |
| *Persicaria lapathifolia* var. *salicifolia* | 159049 | 83618 | 31141 | 13149 | 38.2 | 36.6 | 41.5 | 33.2 |
| *Persicaria longiseta* | 159256 | 83797 | 31144 | 13171 | 38.2 | 36.5 | 41.4 | 33.2 |
| *Persicaria longiseta* var. *rotundata* 1 | 159401 | 84000 | 31113 | 13175 | 38.2 | 36.6 | 41.5 | 33.2 |
| *Persicaria longiseta* var*. rotundata* 2 | 159436 | 84022 | 31120 | 13174 | 38.2 | 36.5 | 41.5 | 33.2 |
| *Persicaria maackiana* | 160682 | 85422 | 31131 | 12998 | 37.9 | 36.1 | 41.4 | 32.9 |
| *Persicaria maculosa* | 159076 | 83644 | 31141 | 13150 | 38.2 | 36.6 | 41.5 | 33.2 |
| *Persicaria neofiliformis* | 160028 | 84701 | 31096 | 13135 | 37.8 | 36.0 | 41.3 | 32.5 |
| *Persicaria nepalensis* | 157889 | 83443 | 30805 | 12836 | 37.9 | 36.1 | 41.5 | 33.0 |
| *Persicaria orientalis* | 159015 | 83584 | 31139 | 13153 | 38.2 | 36.6 | 41.5 | 33.2 |
| *Persicaria perfoliata* | 160737 | 85440 | 31185 | 12927 | 38.0 | 36.2 | 41.4 | 33.1 |
| *Persicaria posumbu* | 159350 | 83961 | 31108 | 13173 | 38.2 | 36.5 | 41.5 | 33.2 |
| *Persicaria runcinata* | 158985 | 84286 | 30903 | 12893 | 38.0 | 36.1 | 41.4 | 33.4 |
| *Persicaria sagittata* | 160422 | 85068 | 31188 | 12978 | 37.8 | 36.0 | 41.4 | 32.7 |
| *Persicaria senticosa* | 160589 | 85239 | 31216 | 12918 | 38.0 | 36.3 | 41.4 | 33.3 |
| *Persicaria taquetii* | 159374 | 83939 | 31131 | 13173 | 38.2 | 36.5 | 41.5 | 33.2 |
| *Persicaria thunbergii* | 159848 | 84661 | 31070 | 13047 | 38.0 | 36.2 | 41.4 | 33.0 |
| *Persicaria viscofera* | 159621 | 84152 | 31139 | 13191 | 38.2 | 36.6 | 41.4 | 33.2 |
| *Persicaria viscosa* | 159065 | 83631 | 31141 | 13152 | 38.2 | 36.6 | 41.5 | 33.2 |
