# Supplementary material for: Plastid phylogenomics sheds light on divergence time and ecological adaptations of the tribe Persicarieae (Polygonaceae)
Source: Front Plant Sci. 2022 Dec 8;13:1046253. doi: 10.3389/fpls.2022.1046253 (PMC9780030; doi:10.3389/fpls.2022.1046253)
Supplement: Supplementary file 2 [file DataSheet_2.zip › Table 4.DOCX]

**Table S4** SSR distribution in the 59 Persicarieae plastomes.

| ***Species*** | **Total SSRs** | **Compound SSRs** | **A/T** | | **C/G** | | **AG/CT** | **AT/TA** | | **AAT/ATT** | | | | **LSC** | **SSC** | **IR_a_** | **IR_b_** |
| --- | --- | --- | --- | --- | --- | --- | --- | --- | --- | --- | --- | --- | --- | --- | --- | --- | --- |
|  |  |  | **A** | **T** | **C** | **G** | **TC** | **AT** | **TA** | **ATA** | **ATT** | **TAT** | **TTA** |  |  |  |  |
| *Bistorta amplexicaulis* | 41 | 3 | 21 | 16 | - | - | - | 3 |  | - | - | 1 | - | 31 | 4 | 3 | 3 |
| *Bistorta coriacea* | 43 | 1 | 20 | 16 | 2 | - | - | 3 | 1 | - | - | 1 | - | 33 | 4 | 3 | 3 |
| *Bistorta emodi* | 50 | 2 | 17 | 23 | 1 | 2 | - | 4 | 3 | - | - | - | - | 41 | 1 | 4 | 4 |
| *Bistorta macrophylla* | 43 | 2 | 19 | 17 | 2 | - | - | 3 | 1 | - | - | 1 | - | 32 | 5 | 3 | 3 |
| *Bistorta milletii* | 39 | 5 | 18 | 16 | 2 | - | - | 3 |  | - | - | - | - | 27 | 6 | 3 | 3 |
| *Bistorta ochotensis* | 37 | 2 | 16 | 14 | 2 | 1 | - | 2 | 2 | - | - | - | - | 29 | 2 | 3 | 3 |
| *Bistorta officinalis* | 37 | 3 | 17 | 14 | 1 | - | - | 3 | 2 | - | - | - | - | 27 | 4 | 3 | 3 |
| *Bistorta paleaceum* | 44 | 1 | 19 | 17 | 2 | - | - | 4 | 1 | - | - | 1 | - |  |  |  |  |
| *Bistorta sinomontana* | 37 | 3 | 20 | 13 | - | - | - | 3 |  | - | - | 1 | - | 27 | 4 | 3 | 3 |
| *Bistorta suffulta* | 45 | 2 | 20 | 17 | 2 | 1 | - | 3 | 1 | - | - | 1 | - | 35 | 4 | 3 | 3 |
| *Bistorta vivipara* | 41 | 2 | 18 | 16 | 2 | - | - | 3 | 1 | - | - | 1 | - | 31 | 4 | 3 | 3 |
| *Koenigia ajanense* | 45 | 3 | 16 | 24 | 2 | 1 | - | 2 |  | - | - | - | - | 27 | 6 | 6 | 6 |
| *Koenigia alpinum* | 48 | 2 | 16 | 25 | 2 | 3 | - | 2 |  | - | - | - | - | 30 | 6 | 6 | 6 |
| *Koenigia campanulata* var. *fulvida* | 41 | 2 | 19 | 16 | 2 | 2 | - | 1 | 1 | - | - | - | - | 22 | 3 | 8 | 8 |
| *Koenigia cyanandra* 1 | 34 | 3 | 12 | 14 | 1 | 4 | - | 1 | 1 | - | - | 1 | - | 23 | 3 | 4 | 4 |
| *Koenigia cyanandra* 2 | 34 | 3 | 12 | 14 | 1 | 4 | - | 1 | 1 | - | - | 1 | - | 23 | 3 | 4 | 4 |
| *Koenigia delicatula* 1 | 25 | - | 7 | 13 | 2 | - | - | 2 | 1 | - | - | - | - | 11 | 2 | 6 | 6 |
| *Koenigia delicatula* 2 | 26 | - | 10 | 11 | 2 | - | - | 2 | 1 | - | - | - | - | 12 | 2 | 6 | 6 |
| *Koenigia divaricata* | 47 | 1 | 16 | 26 | 1 | 2 | - | 2 |  | - | - | - | - | 29 | 6 | 6 | 6 |
| *Koenigia forrestii* | 38 | 1 | 14 | 20 | 2 | 2 | - |  |  | - | - | - | - | 21 | 7 | 5 | 5 |
| *Koenigia islandica* | 33 | - | 10 | 14 | 2 | 3 | - | 1 | 3 | - | - | - | - | 22 | 5 | 3 | 3 |
| *Koenigia lichiangensis* | 46 | 2 | 23 | 17 | 2 | 2 | - | 1 | 1 | - | - | - | - | 24 | 4 | 9 | 9 |
| *Koenigia mollis* | 45 | 1 | 20 | 21 | 1 | 3 | - |  |  | - | - | - | - | 30 | 3 | 6 | 6 |
| *Koenigia mollis* var. *rudis* | 42 | 1 | 19 | 21 | 1 | 1 | - |  |  | - | - | - | - | 27 | 3 | 6 | 6 |
| *Koenigia nepalensis* | 34 | - | 13 | 19 | 1 | 1 | - |  |  | - | - | - | - | 19 | 5 | 5 | 5 |
| *Persicaria amphibia* 1 | 26 | 1 | 10 | 12 | - | 1 | - |  | 1 | - | 1 |  | 1 | 17 | 1 | 4 | 4 |
| *Persicaria amphibia* 2 | 26 | 1 | 10 | 12 | - | 1 | - |  | 1 | - | 1 |  | 1 | 17 | 1 | 4 | 4 |
| *Persicaria bungeana* | 32 | - | 15 | 14 | - | - | - | 1 | 2 | - | - | - | - | 20 | 4 | 4 | 4 |
| *Persicaria capitata* | 33 | 2 | 15 | 13 | - | 3 | - | 1 | 1 | - | - | - | - | 22 | 1 | 5 | 5 |
| *Persicaria chinense* var. *paradoxum* | 39 | 2 | 18 | 16 | 2 | 2 | - |  | 1 | - | - | - | - | 24 | 1 | 7 | 7 |
| *Persicaria dissitiflora* | 28 | 1 | 10 | 15 | 1 | 1 | - |  | 1 | - | - | - | - | 21 | 1 | 3 | 3 |
| *Persicaria filiformis* | 32 | 1 | 15 | 14 | 1 | 2 | - |  |  | - | - | - | - | 23 | 1 | 4 | 4 |
| *Persicaria neofiliforme* | 33 | 1 | 16 | 15 | - | - | - | 1 | 1 | - | - | - | - | 24 | 1 | 4 | 4 |
| *Persicaria foliosa* | 25 | 1 | 10 | 14 | - | 1 | - |  |  | - | - | - | - | 19 | - | 3 | 3 |
| *Persicaria glabra* | 37 | 2 | 16 | 17 | - | 1 | - | 1 | 2 | - | - | - | - | 24 | 1 | 6 | 6 |
| *Persicaria glacialis* | 41 | 2 | 20 | 19 | - | 1 | - | 1 |  | - | - | - | - | 28 | 1 | 6 | 6 |
| *Persicaria hastatosagittata* | 44 | 4 | 16 | 20 | 2 | 2 | - |  | 2 | - | 2 | - | - | 32 | 4 | 4 | 4 |
| *Persicaria hydropiper* | 28 | - | 14 | 14 | - | - | - |  |  | - | - | - | - | 18 | 2 | 4 | 4 |
| *Persicaria japonica* 1 | 23 | - | 9 | 14 | - | - | - |  |  | - | - | - | - | 16 | 1 | 3 | 3 |
| *Persicaria japonica* 2 | 23 | - | 9 | 14 | - | - | - |  |  | - | - | - | - | 16 | 1 | 3 | 3 |
| *Persicaria kawagoeana* | 27 | 1 | 11 | 16 | - | - | - |  |  | - | - | - | - | 20 | 1 | 3 | 3 |
| *Persicaria lapathifolia* | 38 | 1 | 16 | 18 | - | 1 | - | 1 | 2 | - | - | - | - | 25 | 1 | 6 | 6 |
| *Persicaria lapathifolia* var. *salicifolia* | 38 | 1 | 15 | 18 | 1 | 1 | - | 1 | 2 | - | - | - | - | 25 | 1 | 6 | 6 |
| *Persicaria longiseta* | 26 | 2 | 11 | 14 | - | - | - |  | 1 | - | - | - | - | 18 | - | 4 | 4 |
| *Persicaria longiseta* var. *rotundata* 1 | 29 | 2 | 13 | 15 | - | - | - | 1 |  | - | - | - | - | 21 | - | 4 | 4 |
| *Persicaria longiseta* var. *rotundata* 2 | 27 | 1 | 12 | 14 | - | - | - | 1 |  | - | - | - | - | 19 | - | 4 | 4 |
| *Persicaria maackiana* | 37 | 1 | 16 | 19 | - | 1 | - |  | 1 | - | - | - | - | 27 | 2 | 4 | 4 |
| *Persicaria maculosa* | 38 | 2 | 14 | 19 | 1 | 1 | - | 1 | 2 | - | - | - | - | 24 | 2 | 6 | 6 |
| *Persicaria nepalensis* | 40 | 3 | 17 | 19 | 1 | 3 | - |  |  | - | - | - | - | 25 | 1 | 7 | 7 |
| *Persicaria orientalis* | 39 | 1 | 15 | 19 | 2 | - | - | 2 | 1 | - | - | - | - | 29 | 2 | 4 | 4 |
| *Persicaria perfoliata* | 28 | 1 | 10 | 14 | 2 | 1 | - | 1 |  | - | - | - | - | 20 | - | 4 | 4 |
| *Persicaria posumbu* | 30 | 2 | 14 | 14 | - | - | - | 1 | 1 | - | - | - | - | 19 | 1 | 5 | 5 |
| *Persicaria runcinata* | 33 | 2 | 15 | 13 | 2 | 2 | - |  | 1 | - | - | - | - | 21 | - | 6 | 6 |
| *Persicaria sagittata* | 40 | 2 | 18 | 15 | 2 | - | 1 | 2 | 1 | 1 | - | - | - | 26 | 2 | 6 | 6 |
| *Persicaria senticosa* | 30 | 2 | 12 | 16 | - | - | - | 1 | 1 | - | - | - | - | 24 | - | 3 | 3 |
| *Persicaria taquetii* | 28 | 1 | 10 | 17 | - | - | - | 1 |  | - | - | - | - | 19 | 1 | 4 | 4 |
| *Persicaria thunbergii* | 34 | 1 | 13 | 18 | - | 1 | - | 1 | 1 | - | - | - | - | 26 | - | 4 | 4 |
| *Persicaria viscofera* | 29 | 1 | 11 | 18 | - | - | - |  |  | - | - | - | - | 20 | 1 | 4 | 4 |
| *Persicaria viscosa* | 37 | 1 | 17 | 18 | - | - | - | 1 | 1 | - | - | - | - | 23 | 2 | 6 | 6 |
