# Supplementary material for: Plastid phylogenomics sheds light on divergence time and ecological adaptations of the tribe Persicarieae (Polygonaceae)
Source: Front Plant Sci. 2022 Dec 8;13:1046253. doi: 10.3389/fpls.2022.1046253 (PMC9780030; doi:10.3389/fpls.2022.1046253)
Supplement: Supplementary file 2 [file DataSheet_2.zip › Table 5.DOCX]

**Table S5** Repeat sequence analysis of 59 Persicarieae plastomes.

| **Species** | **Forward** | **Reverse** | **Complement** | **Palindromic** | **Total** |
| --- | --- | --- | --- | --- | --- |
| *Bistorta coriacea* | 17 | 2 | 0 | 17 | 36 |
| *Bistorta emodi* | 23 | 1 | 1 | 23 | 48 |
| *Bistorta macrophylla* | 17 | 2 | 0 | 17 | 36 |
| *Bistorta milletii* | 20 | 2 | 0 | 19 | 41 |
| *Bistorta ochotensis* | 20 | 1 | 0 | 18 | 39 |
| *Bistorta officinalis* | 21 | 1 | 0 | 20 | 42 |
| *Bistorta paleaceum* | 17 | 2 | 0 | 17 | 36 |
| *Bistorta sinomontana* | 21 | 2 | 0 | 19 | 42 |
| *Bistorta amplexicaulis* | 23 | 6 | 0 | 19 | 48 |
| *Bistorta suffulta* | 17 | 3 | 0 | 18 | 38 |
| *Bistorta vivipara* | 17 | 1 | 0 | 17 | 35 |
| *Koenigia ajanense* | 17 | 0 | 0 | 17 | 34 |
| *Koenigia alpinum* | 16 | 0 | 0 | 15 | 31 |
| *Koenigia campanulata* var. *fulvida* | 8 | 0 | 0 | 8 | 16 |
| *Koenigia cyanandra* 1 | 8 | 0 | 0 | 8 | 16 |
| *Koenigia cyanandra* 2 | 10 | 0 | 0 | 12 | 22 |
| *Koenigia delicatula* 1 | 8 | 1 | 0 | 4 | 13 |
| *Koenigia delicatula* 2 | 8 | 1 | 0 | 4 | 13 |
| *Koenigia divaricata* | 16 | 0 | 0 | 15 | 31 |
| *Koenigia forrestii* | 11 | 1 | 0 | 13 | 25 |
| *Koenigia islandica* | 13 | 2 | 0 | 13 | 28 |
| *Koenigia lichiangensis* | 10 | 0 | 0 | 7 | 17 |
| *Koenigia mollis* | 12 | 1 | 1 | 13 | 27 |
| *Koenigia mollis* var*. rudis* | 12 | 2 | 1 | 14 | 29 |
| *Koenigia nepalensis* | 11 | 0 | 0 | 13 | 24 |
| *Persicaria amphibia* 1 | 13 | 0 | 0 | 13 | 26 |
| *Persicaria amphibia* 2 | 13 | 0 | 0 | 13 | 26 |
| *Persicaria bungeana* | 9 | 0 | 0 | 9 | 18 |
| *Persicaria capitata* | 14 | 0 | 0 | 13 | 27 |
| *Persicaria chinense* var. *paradoxum* | 14 | 0 | 3 | 14 | 31 |
| *Persicaria dissitiflora* | 16 | 0 | 0 | 12 | 28 |
| *Persicaria filiformis* | 12 | 0 | 0 | 12 | 24 |
| *Persicaria neofiliforme* | 12 | 0 | 0 | 12 | 24 |
| *Persicaria foliosa* | 9 | 0 | 0 | 9 | 18 |
| *Persicaria glabra* | 11 | 0 | 0 | 10 | 21 |
| *Persicaria glacialis* | 14 | 0 | 0 | 17 | 31 |
| *Persicaria hastatosagittata* | 15 | 4 | 0 | 15 | 34 |
| *Persicaria hydropiper* | 12 | 0 | 0 | 11 | 23 |
| *Persicaria japonica* 1 | 9 | 0 | 0 | 9 | 18 |
| *Persicaria japonica* 2 | 12 | 0 | 0 | 9 | 21 |
| *Persicaria kawagoeana* | 9 | 0 | 0 | 9 | 18 |
| *Persicaria lapathifolia* | 9 | 0 | 0 | 10 | 19 |
| *Persicaria lapathifolia* var. *salicifolia* | 9 | 0 | 0 | 10 | 19 |
| *Persicaria longiseta* | 14 | 0 | 0 | 13 | 27 |
| *Persicaria longiseta* var. *rotundata* 1 | 11 | 0 | 0 | 11 | 22 |
| *Persicaria longiseta* var. *rotundata* 2 | 11 | 0 | 0 | 11 | 22 |
| *Persicaria maackiana* | 12 | 2 | 0 | 13 | 27 |
| *Persicaria maculosa* | 11 | 0 | 0 | 10 | 21 |
| *Persicaria nepalensis* | 14 | 0 | 0 | 17 | 31 |
| *Persicaria orientalis* | 13 | 0 | 0 | 10 | 23 |
| *Persicaria perfoliata* | 14 | 0 | 0 | 13 | 27 |
| *Persicaria posumbu* | 9 | 0 | 0 | 9 | 18 |
| *Persicaria runcinata* | 13 | 0 | 1 | 14 | 28 |
| *Persicaria sagittata* | 17 | 0 | 0 | 17 | 34 |
| *Persicaria senticosa* | 17 | 0 | 0 | 13 | 30 |
| *Persicaria taquetii* | 11 | 0 | 0 | 11 | 22 |
| *Persicaria thunbergii* | 16 | 0 | 0 | 11 | 27 |
| *Persicaria viscofera* | 16 | 0 | 0 | 11 | 27 |
| *Persicaria viscosa* | 11 | 0 | 0 | 10 | 21 |

| **Species** | **30** | **31** | **32** | **33** | **34** | **35** | **36** | **37** | **38** | **39** | **40** | **41** | **42** | **43** | **44** | **46** | **47** | **48** | **49** | **50** | **51** | **52** | **53** | **55** | **57** | **60** |
| --- | --- | --- | --- | --- | --- | --- | --- | --- | --- | --- | --- | --- | --- | --- | --- | --- | --- | --- | --- | --- | --- | --- | --- | --- | --- | --- |
| *Bistorta coriacea* | 9 | 6 | 1 | 1 | 6 | 1 |  |  |  | 3 |  |  | 2 | 3 |  |  | 4 |  |  |  |  |  |  |  |  |  |
| *Bistorta emodi* | 11 | 6 | 2 | 5 | 5 | 1 | 1 | 1 | 4 | 3 |  |  | 2 | 3 |  |  | 4 |  |  |  |  |  |  |  |  |  |
| *Bistorta macrophylla* | 9 | 6 | 1 | 1 | 6 | 1 |  |  |  | 3 |  |  | 2 | 3 |  |  | 4 |  |  |  |  |  |  |  |  |  |
| *Bistorta milletii* | 9 | 6 | 1 | 2 | 6 | 1 |  |  |  | 3 |  |  | 2 | 3 |  |  | 4 |  |  |  |  |  |  |  |  |  |
| *Bistorta ochotensis* | 13 | 4 | 1 | 1 | 6 |  |  |  |  | 3 | 1 |  | 3 | 3 |  |  | 4 |  |  |  |  |  |  |  |  |  |
| *Bistorta officinalis* | 16 | 4 | 1 | 1 | 6 |  |  |  | 1 | 3 | 1 |  | 2 | 3 |  |  | 4 |  |  |  |  |  |  |  |  |  |
| *Bistorta paleaceum* | 9 | 6 | 1 | 1 | 6 | 1 |  |  |  | 3 |  |  | 2 | 3 |  |  | 4 |  |  |  |  |  |  |  |  |  |
| *Bistorta sinomontana* | 11 | 5 | 1 | 1 | 6 |  |  |  |  | 3 |  |  | 6 | 3 |  |  | 4 |  |  |  | 1 |  |  |  |  |  |
| *Bistorta speciosa* | 11 | 5 | 1 | 3 | 6 | 2 | 1 |  |  | 3 |  | 1 | 6 | 3 |  |  | 4 |  |  |  | 1 | 1 |  |  |  |  |
| *Bistorta suffulta* | 9 | 5 | 2 | 1 | 6 | 2 | 1 |  |  | 3 |  |  | 2 | 3 |  |  | 4 |  |  |  |  |  |  |  |  |  |
| *Bistorta vivipara* | 9 | 5 | 1 | 1 | 6 | 1 |  |  |  | 3 |  |  | 2 | 3 |  |  | 4 |  |  |  |  |  |  |  |  |  |
| *Koenigia ajanense* | 6 | 8 | 1 | 4 | 1 | 4 |  | 4 |  | 3 |  |  | 2 |  |  |  |  |  |  |  |  |  | 1 |  |  |  |
| *Koenigia alpinum* | 5 | 6 | 1 | 4 | 1 | 4 |  | 4 |  | 3 |  |  | 2 |  |  |  |  |  |  |  |  | 1 |  |  |  |  |
| *Koenigia campanulata* var*. fulvida* | 7 | 1 | 4 | 1 | 1 |  |  |  |  |  |  |  | 2 |  |  |  |  |  |  |  |  |  |  |  |  |  |
| *Koenigia cyanandra* 1 | 7 | 1 | 4 | 1 | 1 |  |  |  |  |  |  |  | 2 |  |  |  |  |  |  |  |  |  |  |  |  |  |
| *Koenigia cyanandra* 2 | 7 | 1 | 2 |  | 5 | 1 |  | 1 |  | 3 |  |  | 2 |  |  |  |  |  |  |  |  |  |  |  |  |  |
| *Koenigia delicatula* 1 | 4 | 2 | 1 |  | 4 |  |  |  |  | 2 |  |  |  |  |  |  |  |  |  |  |  |  |  |  |  |  |
| *Koenigia delicatula* 2 | 4 | 1 | 1 |  | 4 | 1 |  |  |  | 2 |  |  |  |  |  |  |  |  |  |  |  |  |  |  |  |  |
| *Koenigia divaricata* | 5 | 6 | 1 | 4 | 1 | 4 |  | 4 |  | 3 |  |  | 2 |  |  |  |  |  |  |  |  | 1 |  |  |  |  |
| *Koenigia forrestii* | 8 | 2 | 1 | 2 | 5 | 1 |  |  |  | 3 | 1 |  | 2 |  |  |  |  |  |  |  |  |  |  |  |  |  |
| *Koenigia islandica* | 8 | 3 | 2 | 1 | 4 | 2 | 1 |  | 1 | 3 | 1 |  | 2 |  |  |  |  |  |  |  |  |  |  |  |  |  |
| *Koenigia lichiangensis* | 7 | 1 |  | 1 | 1 | 4 |  |  |  |  |  |  | 2 |  |  |  |  |  |  |  | 1 |  |  |  |  |  |
| *Koenigia mollis* | 4 | 2 | 6 | 1 | 1 | 5 |  |  |  | 3 |  | 2 | 2 | 1 |  |  |  |  |  |  |  |  |  |  |  |  |
| *Koenigia mollis* var*. rudis* | 5 | 3 | 6 | 1 | 1 | 5 |  |  |  | 3 |  | 2 | 2 | 1 |  |  |  |  |  |  |  |  |  |  |  |  |
| *Koenigia nepalensis* | 6 | 2 | 1 |  | 5 | 1 |  |  |  | 3 | 1 |  | 2 |  |  |  |  |  | 3 |  |  |  |  |  |  |  |
| *Persicaria amphibia* 1 | 6 | 3 | 1 |  | 5 | 5 |  |  |  | 3 |  |  | 2 |  |  |  |  | 1 |  |  |  |  |  |  |  |  |
| *Persicaria amphibia* 2 | 6 | 3 | 1 |  | 5 | 5 |  |  |  | 3 |  |  | 2 |  |  |  |  | 1 |  |  |  |  |  |  |  |  |
| *Persicaria bungeana* | 4 | 1 | 2 |  | 4 | 4 |  |  |  |  |  |  | 2 |  |  |  |  | 1 |  |  |  |  |  |  |  |  |
| *Persicaria capitata* | 5 | 3 | 3 |  | 4 |  |  |  |  | 3 |  |  | 5 |  | 1 |  | 1 |  | 1 |  |  |  | 1 |  |  |  |
| *Persicaria chinense* var*. paradoxum* | 8 | 4 | 3 |  | 4 |  |  |  |  | 3 |  |  | 5 |  | 1 |  | 1 |  |  |  | 1 |  | 1 |  |  |  |
| *Persicaria dissitiflora* | 8 | 2 | 2 |  | 4 | 4 |  |  |  | 3 |  |  | 3 |  |  |  |  |  | 1 |  |  |  |  | 1 |  |  |
| *Persicaria filiformis* | 6 | 2 | 2 |  | 4 | 4 |  |  |  | 3 | 1 |  | 2 |  |  |  |  |  |  |  |  |  |  |  |  |  |
| *Persicaria neofiliforme* | 6 | 2 | 2 |  | 4 | 4 |  |  |  | 3 |  |  | 2 |  |  |  |  |  |  | 1 |  |  |  |  |  |  |
| *Persicaria foliosa* | 4 | 1 | 1 |  | 4 | 4 | 1 |  |  |  |  |  | 2 |  |  |  |  | 1 |  |  |  |  |  |  |  |  |
| *Persicaria glabra* | 4 | 2 | 2 |  | 4 | 4 |  |  |  | 1 |  |  | 2 |  |  |  | 1 | 1 |  |  |  |  |  |  |  |  |
| *Persicaria glacialis* | 10 | 3 | 3 |  | 4 |  |  |  |  | 3 |  |  | 5 |  | 1 |  | 1 |  |  |  |  |  | 1 |  |  |  |
| *Persicaria hastatosagittata* | 7 | 4 | 2 | 2 | 5 | 4 |  | 1 | 2 | 3 |  |  | 2 |  |  |  | 1 | 1 |  |  |  |  |  |  |  |  |
| *Persicaria hydropiper* | 4 | 1 | 2 |  | 4 | 4 |  |  | 1 |  |  |  | 2 |  |  |  | 4 | 1 |  |  |  |  |  |  |  |  |
| *Persicaria japonica* 1 | 4 | 1 | 2 |  | 4 | 4 |  |  |  |  |  |  | 2 |  |  |  |  | 1 |  |  |  |  |  |  |  |  |
| *Persicaria japonica* 2 | 4 | 1 | 2 |  | 4 | 4 |  |  |  |  |  |  | 2 |  |  |  |  | 1 |  |  |  |  |  |  |  |  |
| *Persicaria kawagoeana* | 4 | 1 | 1 |  | 4 | 4 | 1 |  |  |  |  |  | 2 |  |  |  |  | 1 |  |  |  |  |  |  |  |  |
| *Persicaria lapathifolia* | 4 | 1 | 2 |  | 4 | 4 |  |  |  |  |  |  | 2 |  |  |  | 1 | 1 |  |  |  |  |  |  |  |  |
| *Persicaria lapathifolia* var*. salicifolia* | 4 | 1 | 2 |  | 4 | 4 |  |  |  |  |  |  | 2 |  |  |  | 1 | 1 |  |  |  |  |  |  |  |  |
| *Persicaria longiseta* | 5 | 1 | 2 |  | 4 | 4 |  |  |  | 4 |  |  | 2 |  |  |  |  | 1 |  |  |  |  |  |  | 4 |  |
| *Persicaria longiseta* var*. rotundata* 1 | 4 | 1 | 2 |  | 4 | 4 |  |  |  | 4 |  |  | 2 |  |  |  |  | 1 |  |  |  |  |  |  |  |  |
| *Persicaria longiseta* var*. rotundata* 2 | 4 | 1 | 2 |  | 4 | 4 |  |  |  | 4 |  |  | 2 |  |  |  |  | 1 |  |  |  |  |  |  |  |  |
| *Persicaria maackiana* | 6 | 1 | 1 |  | 5 | 5 | 1 |  |  | 2 |  |  | 2 |  | 1 |  |  | 1 |  |  | 2 |  |  |  |  |  |
| *Persicaria maculosa* | 4 | 2 | 2 |  | 4 | 4 |  |  |  | 1 |  |  | 2 |  |  |  | 1 | 1 |  |  |  |  |  |  |  |  |
| *Persicaria nepalensis* | 6 | 5 | 3 |  | 4 | 4 |  |  |  | 3 |  |  | 5 |  |  |  | 1 |  |  |  |  |  |  |  |  |  |
| *Persicaria orientalis* | 4 | 2 | 2 |  | 4 | 4 |  |  |  | 1 |  |  | 2 |  |  |  | 2 | 1 |  |  |  |  |  |  |  | 1 |
| *Persicaria perfoliata* | 8 | 1 | 2 |  | 4 | 5 |  |  |  | 3 |  |  | 2 | 1 |  |  |  |  |  |  | 1 |  |  |  |  |  |
| *Persicaria posumbu* | 4 | 1 | 2 |  | 4 | 4 |  |  |  |  |  |  | 2 |  |  |  |  | 1 |  |  |  |  |  |  |  |  |
| *Persicaria runcinata* | 6 | 4 | 3 |  | 4 |  |  |  |  | 3 |  |  | 5 |  | 1 |  | 1 |  |  |  |  |  | 1 |  |  |  |
| *Persicaria sagittata* | 13 | 1 | 1 | 1 | 6 | 5 |  |  |  | 3 |  |  | 2 | 1 | 1 |  |  |  |  |  |  |  |  |  |  |  |
| *Persicaria senticosa* | 8 |  | 2 |  | 4 | 5 |  |  |  | 3 |  |  | 2 | 1 |  | 4 |  |  |  |  | 1 |  |  |  |  |  |
| *Persicaria taquetii* | 4 | 1 | 2 |  | 4 | 4 |  |  |  | 4 |  |  | 2 |  |  |  |  | 1 |  |  |  |  |  |  |  |  |
| *Persicaria thunbergii* | 8 | 1 | 1 | 1 | 5 | 4 | 1 |  |  | 2 |  | 1 | 2 |  |  |  |  |  | 1 |  |  |  |  |  |  |  |
| *Persicaria viscofera* | 8 | 1 | 1 | 1 | 5 | 4 | 1 |  |  | 2 |  | 1 | 2 |  |  |  |  |  | 1 |  |  |  |  |  |  |  |
| *Persicaria viscosa* | 4 | 2 | 2 |  | 4 | 4 |  |  |  | 1 |  |  | 2 |  |  |  | 1 | 1 |  |  |  |  |  |  |  |  |
